# Two predators, one prey model that integrates the effect of supplementary food resources due to one predator's kleptoparasitism under the possibility of retribution by the other predator

**DOI:** 10.1016/j.heliyon.2024.e28940

**Published:** 2024-04-02

**Authors:** Debasish Bhattacharjee, Dipam Das, Santanu Acharjee, Tarini Kumar Dutta

**Affiliations:** a Department of Mathematics, Gauhati University, Assam, India; b Department of Mathematics, Assam Don Bosco University, Assam, India

**Keywords:** 92D40, 92D25, 34D20, 34C23, Predator–prey, Kleptoparasitism, Inter-specific competition, Stability, Bifurcation

## Abstract

In ecology, foraging requires animals to expend energy in order to obtain resources. The cost of foraging can be reduced through kleptoparasitism, the theft of a resource that another individual has expended effort to acquire. Thus, kleptoparasitism is one of the most significant feeding techniques in ecology. The phenomenon of kleptoparasitism has garnered significant attention from scholars due to its substantial impact on the food chain. However, the proportionate amount of mathematical modelling to facilitate the analysis has made limited progress in the literature. This circumstance motivated us to develop mathematical models that could explain the population dynamics of the prey-predator food chain. This study explores a scenario with two predators and one prey, where one predator is a kleptoparasite and the other is a host. The energy depletion caused by the predator's counterattack subsequent to kleptoparasitism, notwithstanding the nonlethal nature of this antagonism, is an additional component incorporated into this model. It has been suggested that biologically viable equilibria must meet certain parametric conditions in order to exist and to be stable both locally and globally. This article delves deeply into the occurrences of various one-parametric bifurcations, such as saddle-node bifurcation, transcritical bifurcation, and Hopf bifurcation, as well as two-parametric bifurcations, such as Bautin bifurcation. A subcritical Hopf bifurcation happens when the growth rate of the first predator is relatively low, while a supercritical Hopf bifurcation occurs when the growth rate of the first predator is quite large, allowing for the coexistence of all three species. Numerical simulations have been conducted to validate our theoretical findings.

## 1 Introduction

Predator-prey relationships are a pervasive and diverse natural phenomenon that exerts a significant influence on population dynamics. As such, ecologists have devoted considerable time and resources to their study, with mathematical modelling being a valuable tool for understanding the underlying rationale and strategies that govern these interactions. The study of the dynamics of predator-prey systems dates back to the works of Lotka [1] and Volterra [2]. The Lotka-Volterra model is the fundamental model that elucidates the interaction between two species through a set of non-linear first-order ordinary differential equations. In order to establish the validity of modelling expressions in comparison to reality, numerous modifications have been offered since then to account for a range of ecological phenomena. Despite the fact that numerous ecological models have been developed and have significantly aided our understanding of how prey and predator interactions operate within a system, numerous ecological systems are emerging that do not fall within the collection of mathematical transformations of prey-predator ecological systems. Starting with a simple one-prey, one-predator interaction environment, mathematical models are evolving to incorporate various ecological complexities such as the allee effect [3], [4], [5], [6], fear effect [7], [8], [9], [10], memory effect [6], [9], [11], additional food for predator [12], [13], and others, which exist even in ostensibly simple ecosystems and are a recent area of interest.

The addition of additional species or population divisions to the simple prey-predator system framework and the quantification of the qualitative complexity of the ecosystem constitute a second avenue for the advancement of mathematical modelling that is consistent with reality. Numerous academicians have devised mathematical models depicting interactions between two predators and a single prey [14], [15], [16], [17], [18], [19], [20], [21], [22], [23], [24], two prey and a single predator [7], [25], two predators and two prey species [26], [27], etc. Such expansions might offer insight on the intricacy of species relationships outside of the usual prey-predator conflict, in addition to outlining other ecological complications. In this endeavour, we incorporate a model with two predators and one prey, enriched with the complex interactions of prey-predator and predator-predator.

Prey-predator interaction and predator-predator interaction are the principal kinds of direct interactions that transpire between populations in ecological systems when two predator species coexist with the same prey species, and the heart of the dynamics of the ecosystem is the prey-predator interaction or functional response. The term “functional response of the predator” refers to the rate at which a predator consumes its prey. It is one of the important prey-predator interactions that is responsible for the deaths of both prey and sometimes predator populations. The typical number of prey each predator kills per unit of time serves as a gauge for this rate. Various functional responses have been established in the theoretical ecology literature. In a scholarly article [28], Holling discussed three different functional responses - Holling Type-I, Holling Type-II, and Holling Type-III - to illustrate the impacts of different types of predation. There are numerous other types of functional responses, each with its own distinctive properties [22], [28], [29], [30], [31], [32]. The Holling type-I functional response, which predicts a linear increase, is utilised in this paper. This implies that the time needed for a predator to process a food item is minimal, or that the act of consuming does not impede the predator's ability to forage for additional food. Scholars [5], [18], [33], [34] have examined prey-predator models that exhibit diverse functional responses.

When two predator species subsist off of the same prey, it is impossible to disregard their competition for resources, as competition for resources is common in nature and society [21]. Competition constitutes one of the most important predator-prey interactions. In conformance with this concept, numerous researchers [18], [19], [23], [35] have considered a three-species predator-prey system in which the two predators compete for the same prey. Our model accounts for the interspecific animosity between the two predator species.

This research investigates another type of predator-predator interaction found in nature: “kleptoparasitism”. Although kleptoparasitism is one of the most prevalent foraging strategies in nature for procuring scarce resources and many animals rely heavily on it for subsistence, it has received little consideration in the construction and analysis of predator-prey population models. Although kleptoparasitism has been implicitly incorporated into a small number of mathematical models of ecosystems, for instance, many ecologists [51], [52], [53], [54] discussed a game-theoretical model of kleptoparasitism. Broom et al. [32], [55] discussed a stochastic model of kleptoparasitism. But in these models, ecologists have focused only on the kleptoparasitism scenario, but the whole population dynamics in the long term are not shown. In a recent study, Focardi et al. [37] explored the impact of scavenging on predator and multiprey dynamics in the northern Apennine system in Italy. The study by Materassi et al. [36] is pioneering in incorporating kleptoparasitism into trophic web models to elucidate long-term population dynamics. Aside from these two literary works, the authors are not aware of any other predator-prey models delving into the kleptoparasitism effect in long-term population dynamics.

Kleptoparasitism is a feeding technique in which one animal steals resources from another. Rothschild and Clay [38] coined the word “clepto-parasitism” or “kleptoparasitism” to refer to the act of one species' members stealing food from the other's members of a different species. The same behaviour is also referred to as ‘piracy’ by many authors [39], [40], as well as ‘food parasitism’ [41], and ‘pilfering’ [42]. This feeding strategy becomes more important when food is scarce in nature or it takes too much energy to hunt. Kleptoparasites acquire sustenance without the need to invest time and energy in hunting and killing prey. Kleptoparasitism enables animals to redirect their time and energy from foraging towards activities like finding mates or evading predators [43]. Thus, kleptoparasitism boosts the growth rate of that species. Many examples of kleptoparasitism are found in nature. For instance, kleptoparasitism in ant-eating spiders belonging to the genus Zodarion. During the field investigation, researchers [44] observed that adult females constantly engaged in active hunting, while adult males shifted to kleptoparasitism to increase their chances of mating. Exploring the phenomenon of kleptoparasitism reveals its prevalence among various bird species, particularly within specific families of seabirds such as Chionididae (sheathbills), Stercoraridae (skuas), Fregatidae (frigatebirds), and Laridae (gulls and terns). For example, frigatebirds and skuas acquire a significant amount of their food through kleptoparasitism of heterospecifics [45].

Spotted hyenas (Crocuta crocuta) and lions (Panthera leo) are potentially serious competitors for food, as they show a significant overlap in their diet [46], [47]. Both species are subject to kleptoparasitism from each other [48], [49], [50]. We address the effects of kleptoparasitism on two predators and one prey in our model, which further incorporates the post-kleptoparasitism repercussions in terms of energy loss from the predator's counter-attack, despite the fact that this aggression is not fatal. Transformation of the immensely natural post-kleptoparasitism effect into a mathematical form and incorporation into a mathematical model to demonstrate its long-term influences on population dynamics is coined for the first time, to the best of the author's knowledge.

Risks are associated with kleptoparasitism. The reaction of the food owner to a kleptoparasite differs depending on its dominance status. In many cases, it can be seen that the food owner engages in a counter-attack on the Kleptoparasite [56]. We can find evidence of a counter-attack during kleptoparasitism in nature. Black-headed gulls, scientifically known as Larus ridibundus, depend on stealth and quickness to pilfer prey from more powerful species. Black-headed gulls are seen to skillfully snatch a worm in mid-air from the beak of a curlew, scientifically known as Numenius arquata and manage to evade the counter-attack of the surprised victim [57]. Experimentally, Rooks (Corvus frugilegus) are found to be especially susceptible to kleptoparasitism. Although sometimes Rooks make counter-attacks on the kleptoparasite [56]. The kleptoparasite can mitigate the impact of these counter-attacks by engaging more individuals in kleptoparasitism because the effects of the reprisal can be distributed among the kleptoparasites.

In endorsement of the aforementioned natural phenomena, we analyse a mathematical model with two predators competing for the same prey, where one predator species employs kleptoparasitism with the possibility of a counter-attack. The model pertains to a significant phenomenon, kleptoparasitism, which has been validated as such through its comprehensive examination in the literature, yet lags behind in the mathematical analysis of population dynamics. The model describes the effect of kleptoparasitism on the population dynamics of every species using a mathematical model whose structural characteristics distinguish it from the few comparable mathematical models that are known to exist in the literature. In Section 2, the mathematical modelling is presented along with an explanation of its components. Section 3 covers areas such as the solution's positivity and boundedness, local stability, and global stability analysis. The existence of different types of bifurcations and the direction and stability of Hopf bifurcations are demonstrated in Section 4. In Section 5, we validate the results through various numerical simulations, and in Section 6, we derive the necessary conclusions.

## 2 Model construction

Within this section, a mathematical model is developed based on our observations of the phenomenon of kleptoparsitism among predator species. Prior to formulating the model, the following assumptions are established:

1. We consider a scenario wherein two predatory species vie for the same prey. At any given time, *t* and *s* symbolise the population size of the prey species, while $p_{1}$ and $p_{2}$ represent the population sizes of the first and second predator species, respectively.
2. In the absence of the predator, we presume that prey growth follows a logistic pattern due to resource constraints in nature. The ecosystem's carrying capacity is denoted as *k*.
3. We make the assumption that the first predator (with a size of $p_{1}$) consumes the prey (sized *s*) adopting the Holling Type-I functional response. The second predator utilises the identical functional response as the first predator to consume its prey. We hypothesise that the prey is very small in comparison to both predator species; as a result, handling time is minimal, and it therefore qualifies for the Holling type I functional response.
4. Competition arises when the presence of other individuals reduces one's ability to obtain resources. Predator competition can arise when members of the same or different species compete for the same prey. We assume that these two predators are engaged in interspecific competition with each other.
5. It is assumed that both predator species die naturally. Based on the assumptions mentioned earlier, the subsequent model equations are derived:
  (1) $$\frac{ds}{dt}=r_{1}s(1-\frac{s}{k})-sp_{1}r_{2}-sp_{2}r_{3}\;\frac{dp_{1}}{dt}=r_{4}r_{2}sp_{1}-k_{1}p_{1}p_{2}-d_{2}p_{1}\;\frac{dp_{2}}{dt}=r_{5}sp_{2}r_{3}-k_{2}p_{1}p_{2}-d_{3}p_{2}$$
  Hsu et al. [58] investigated this mathematical model and examined the correlation between the coefficient of interference and the outcome of the competition.
6. In addition to (1), we consider that the second predator engages in kleptoparasitism and steals food or resources from the first predator, and apart from that, the second predator also hunts on its prey (of size *s*). So, in this study, the second predator is regarded as the kleptoparasite and the first predator as the host. In kleptoparasitism behaviour, the predator that is the victim of kleptoparasitism (host) faces a heavy loss in their growth rate, while the predator that performs kleptoparasitism (kleptoparasite) enjoys a positive effect on their growth. Let $\psi =\frac{1}{1+ap_{2}}$, where *ψ* denotes the fraction of s killed by per capita of first predator and really eaten by them, i.e., converted into their biomass. So, *ψ*∈ (0,1) and (1-*ψ*) will denote the fraction of s killed by per capita of first predator species but not eaten by them. Kleptoparasitism by the second predator costs per capita of the first predator a fraction (1−ψ) of their food. Here *a* denotes the intensity of kleptoparasitism. Evidently, the following ecological hypotheses are satisfied by the function *ψ*:
  (i) $\lim_{a\to 0}\psi =1$; i.e., if kleptoparsitism intensity of the second predator is negligible then the first predator eats all their kills.
  (ii) $\lim_{a\to \infty }\psi =0$; i.e., every kill of the first predator will be plundered if the second predator's rate of kleptoparasitism rises significantly, leaving nothing for the first predator to imbibe.
  Total food for the first predator by hunting is = $r_{2}sp_{1}$
  Incorporating kleptoparasitism by the second predator, the amount of food for the first predator will be reduced to $\frac{r_{2}sp_{1}}{1+ap_{2}}$, which will be converted to their biomass with a conversion rate of $r_{4}$.
7. The amount of food available for the second predator to consume through kleptoparasitism is $\frac{sp_{1}p_{2}r_{2}a}{1+ap_{2}}$. Let the conversion rate of the food acquired through kleptoparasitism by the second predator species be $r_{7}$. The second predator is considered to be capable of kleptoparasitism either individually or collectively, whereas the first predator pursues it independently. We assume that $r_{7}$ is dependent on the population size of the second predator. This is a consequence of the post-kleptoparasitism effect, which is the first predator's counter-attack or apprehension of one. When the second predator engages in kleptoparasitism over the first predator, there is a possibility of a counter-attack by them due to rage. It is obvious that the second predator can provide a better group defence for their stolen food from the first predator as its size increases. Hence, the remaining amount of stolen food after counter-attack is directly dependent on the population size of the second predator. So we may assume that the conversion rate of the stolen food is dependent on the population size of the second predator.
  There is a possibility that as the second predator's group increases, the concern of the first predator's reprisal diminishes, which could lead to an increase in the conversion rate. According to the aforementioned situation, it is reasonable to infer that the population size of the second predator affects how much of the stolen food is converted. We assume $r_{7}=r_{6}^{\prime }(1+ap_{2})$, where $r_{6}^{\prime }$ is proportionality constant and $\lim_{p_{2}\to 0}r_{7}=r_{6}^{\prime }$. Therefore, the amount $\frac{sp_{1}p_{2}r_{2}a}{1+ap_{2}}$ will get converted into the biomass of the second predator, and the term will convert into $\frac{r_{7}sp_{1}p_{2}r_{2}a}{1+ap_{2}}=r_{6}^{\prime }ar_{2}sp_{1}p_{2}=r_{6}sp_{1}p_{2}$, where $r_{6}=r_{6}^{\prime }ar_{2}$.
8. If the first predator is successful in driving the second predator away from the food by mounting a counter-attack after being kleptoparasitized by the second predator, the stolen food that is recovered from the second predator is assumed not to contribute to its rate of development.

Under these new circumstances, the system (1) changes into the following modified version:

(2) $$\frac{ds}{dt}=r_{1}s(1-\frac{s}{k})-sp_{1}r_{2}-sp_{2}r_{3}\;\frac{dp_{1}}{dt}=\frac{r_{4}r_{2}sp_{1}}{\left(1+ap_{2}\right)}-k_{1}p_{1}p_{2}-d_{2}p_{1}\;\frac{dp_{2}}{dt}=r_{5}sp_{2}r_{3}-k_{2}p_{1}p_{2}-d_{3}p_{2}+r_{6}p_{1}p_{2}s$$

with initial conditions: $s(0)=s^{0}>0,p_{1}(0)=p_{1}^{0}>0$ and $p_{2}(0)=p_{2}^{0}>0$

Here, ‘$r_{1}$’ represents the intrinsic growth rate of the prey species, and ‘$r_{2}$’ and ‘$r_{3}$’ represent the first and second predator's predation rates, respectively. ‘$r_{4}$’ and ‘$r_{5}$’ represent the conversion efficiencies of the first and second predators, respectively, in the event of direct predation. ‘$k_{1}$’ and ‘$k_{2}$’ represent interspecific competition rates for the first and second predators, respectively; ‘$d_{2}$’ and ‘$d_{3}$’ represent the death rates of the first and second predators, respectively. ‘*k*’ is the environmental carrying capacity of prey, and ‘*a*’ is the intensity of kleptoparasitism.

For further simplification of the model (2), we contemplate the following transformations

$$s=Sk,p_{1}=\frac{P_{1}r_{1}}{r_{2}},p_{2}=\frac{P_{2}r_{1}}{r_{3}},T=r_{1}t$$

and the biosystem (2) simplifies to the subsequent system (using t rather than T for notation ease)

(3) $$\frac{dS}{dt}=S(1-S)-SP_{1}-SP_{2}\;\frac{dP_{1}}{dt}=\frac{A_{4}SP_{1}}{\left(A_{1}+P_{2}\right)}-A_{2}P_{1}P_{2}-A_{3}P_{1}\;\frac{dP_{2}}{dt}=A_{5}SP_{2}-P_{1}P_{2}A_{6}-A_{7}P_{2}+A_{8}P_{1}P_{2}S$$

With initial conditions $S(0)=S^{0},P_{1}(0)=P_{1}^{0}$ and $P_{2}(0)=P_{2}^{0}$.

Here $A_{1}=\frac{r_{3}}{ar_{1}}$, $A_{2}=\frac{k_{1}}{r_{3}}$, $A_{3}=\frac{d_{2}}{r_{1}}$, $A_{4}=\frac{r_{2}r_{4}kr_{3}}{ar_{1}^{2}}$, $A_{5}=\frac{r_{5}kr_{3}}{r_{1}}$, $A_{6}=\frac{k_{2}}{r_{2}}$, $A_{7}=\frac{d_{3}}{r_{1}}$, and $A_{8}=\frac{r_{6}k}{r_{2}}$.

## 3 Ecological feasibility and sustainability of the model

The survival of species is an integral component of an ecosystem and is dependent on it directly or indirectly. Consequently, the species cannot expand indefinitely with finite resources. As a result, a model must satisfy both the positivity and boundedness of a species in order to accurately represent a particular ecosystem. In this section, we assess positivity and boundedness to determine ecological feasibility, then the proposed system's equilibrium points (3) and their stability to ensure its long-term viability.

### 3.1 Positivity and boundedness

It is essential to show that the system (3)'s solution is both positive and bounded as it reflects populations. To demonstrate that our current system (3) only yields positive solutions, we can express it in the following manner:

(4) $$\frac{dS}{dt}=S\phi_{1}(S,P_{1},P_{2})\frac{dP_{1}}{dt}=P_{1}\phi_{2}(S,P_{1},P_{2})\frac{dP_{2}}{dt}=P_{2}\phi_{3}(S,P_{1},P_{2})$$

where, $\phi_{1}(S,P_{1},P_{2})=(1-S)-P_{1}-P_{2}$, $\phi_{2}(S,P_{1},P_{2})=\frac{A_{4}S}{\left(A_{1}+P_{2}\right)}-A_{2}P_{2}-A_{3}$, $\phi_{3}(S,P_{1},P_{2})=A_{5}S-P_{1}A_{6}-A_{7}+A_{8}P_{1}S$.

We utilise the following theorem to show that all of system (3)'s solutions are positive.

Theorem 1 *[24]* *Every solution to the given differential equation* $\frac{dX}{dt}=X\xi (X,Y)$ *is guaranteed to be positive.*

Theorem 2 *All solutions to the system* (3) *are consistently positive.*

Proof Theorem 1 gives proof for the theorem since system (3) is stated as system (4). □

Theorem 3 *All the solutions of the system* (3) *are bounded.*

Proof The initial equation of the system representing the prey equation is bounded by the inequality $\frac{dS}{dt}\leq S(1-S)$. By solving the differential inequality, we obtain $\underset{t\to \infty }{\lim\;\sup}\;S(t)\leq 1$ or S(t)≤1.

We construct a function, $D(t)=S+\frac{P_{1}}{A_{9}}+\frac{P_{2}}{A_{5}}$, and then by differentiating it, we obtain

$$\frac{dD}{dt}\leq S(1-S)-P_{1}P_{2}(\frac{A_{2}}{A_{9}}+\frac{A_{10}}{A_{5}})-\frac{A_{3}}{A_{9}}P_{1}-\frac{A_{7}}{A_{5}}P_{2}$$

Let us select *ϕ* such that $\phi <min(A_{3},A_{7})$, which will lead to the modified inequality below

$$\frac{dD}{dt}+\phi D\leq S(1-S+\phi )-P_{1}P_{2}(\frac{A_{2}}{A_{9}}+\frac{A_{10}}{A_{5}})-(\frac{A_{3}}{A_{9}}-\frac{\phi }{A_{9}})P_{1}-(\frac{A_{7}}{A_{5}}-\frac{\phi }{A_{5}})P_{2}$$

which reduces to

$$\frac{dD}{dt}+\phi D\leq S(1-S+\phi )\leq \frac{{\left(1+\phi \right)}^{2}}{4}=\eta$$

Here, $A_{9}=\frac{A_{4}}{A_{1}}$ and $A_{10}=(A_{6}-A_{8})$. Now, by using theory of differential inequality, we get $0<D(t)\leq \frac{\eta }{\phi }(1-e^{-\phi t})+D(0)e^{-\phi t}$and for large value of t, i.e., t → ∞, we have $0<D(t)\leq \frac{\eta }{\phi }$ i.e., does not depend on the initial conditions and therefore, biosystem (3) is bounded. Hence, all solutions of system (3) are contained within the region $\Phi =\left((S,P_{1},P_{2}):0\leq S+\frac{P_{1}}{A_{9}}+\frac{P_{2}}{A_{5}}\leq \frac{\eta }{\phi }+\theta ,\forall \theta >0\right)$. □

### 3.2 Existence of equilibrium points

This section discusses the equilibrium points of system (3) and the conditions necessary for their existence. Six equilibrium points are theoretically defined, but just five of them are ecologically meaningful. They are outlined below:

(a) Extinct equilibrium $E_{0}$ ≡ (0,0,0) always exists without any parametric conditions. It depicts an ecosystem devoid of any species habitats under investigation.
(b) Predator free equilibrium $E_{1}\equiv$ (1,0,0) always exists and it represents the situation of only prey's survival up to the full carrying capacity.
(c) $E_{2}\equiv (B_{1},C_{1},0)$ represents one of the boundary equilibrium points that is free from the second predator. Here, $B_{1}=\frac{A_{1}A_{3}}{A_{4}}$, $C_{1}=\frac{-A_{1}A_{3}+A_{4}}{A_{4}}$ and it exists under the condition $A_{4}>A_{1}A_{3}$. It is this hypothetical situation that explains the eradication of the second predator from the ecosystem.
(d) $E_{3}\equiv (B_{2},0,D_{1})$ represents one of the boundary equilibrium points that is free from the first predator. Here, $B_{2}=\frac{A_{7}}{A_{5}}$ and $D_{1}=\frac{A_{5}-A_{7}}{A_{5}}$, and it exists under the condition $A_{5}>A_{7}$. It is this hypothetical situation that explains the eradication of the first predator from the ecosystem.
(e) The equilibrium point $E_{4}\equiv (0,C_{2},D_{2})$ portrays the prey free scenario in the ecosystem. Here, $C_{2}=-\frac{A_{7}}{A_{6}}$, $D_{2}=-\frac{A_{3}}{A_{2}}$, and obviously it does not exist.
(f) $E^{⁎}\equiv (S^{⁎},P_{1}^{⁎},P_{2}^{⁎})$ represents the interior equilibrium point or coexisting equilibrium point in which all three species coexist.

$E^{⁎}$ is a positive real solution of the system of equations

(5) $$(1-S)-P_{1}-P_{2}=0\;\frac{A_{4}S}{\left(A_{1}+P_{2}\right)}-A_{2}P_{2}-A_{3}=0\;A_{5}S-P_{1}A_{6}-A_{7}+A_{8}P_{1}S=0$$

Using (5) and solving prey nullcline and first predator nullcline for $P_{1}$ and $P_{2}$ and substituting these values in the second nullcline, it is found that S satisfies a polynomial equation of degree 4, which is given by

(6) $$\gamma_{1}S^{⁎4}+\gamma_{2}S^{⁎3}+\gamma_{3}S^{⁎2}+\gamma_{4}S^{⁎}+\gamma_{5}=0$$

Let, $S^{⁎}$ be a positive root of this above polynomial, then $P_{1}^{⁎}=\frac{1}{2}\left(-\frac{\sqrt{A_{1}^{2}A_{2}^{2}-2A_{1}A_{2}A_{3}+4A_{2}A_{4}S^{⁎}+A_{3}^{2}}}{A_{2}}+A_{1}+\frac{A_{3}}{A_{2}}-2S^{⁎}+2\right)$, provided $A_{4}<\frac{A_{2}+A_{1}A_{2}+A_{3}+A_{1}A_{3}-2A_{2}S^{⁎}-A_{1}A_{2}S^{⁎}-A_{3}S^{⁎}+A_{2}S^{⁎2}}{S^{⁎}}$ and $P_{2}^{⁎}=\frac{\sqrt{A_{1}^{2}A_{2}^{2}-2A_{1}A_{2}A_{3}+4A_{2}A_{4}S^{⁎}+A_{3}^{2}}-A_{1}A_{2}-A_{3}}{2A_{2}}$, provided $A_{4}>\frac{A_{1}A_{3}}{S^{⁎}}$.

Here, $\gamma_{1}=4A_{2}^{2}A_{8}^{2}$, $\gamma_{2}=-4A_{2}A_{8}(2A_{2}(A_{5}+A_{6})+((2+A_{1})A_{2}+A_{3}+A_{4})A_{8})$, $\gamma_{3}=4A_{2}(A_{2}{\left(A_{5}+A_{6}\right)}^{2}+(A_{3}A_{5}+2(A_{3}+A_{4})A_{6}+(2+A_{1})A_{2}(A_{5}+2A_{6})+2A_{2}A_{7})A_{8}+(1+A_{1})(A_{2}+A_{3})A_{8}^{2})$, $\gamma_{4}=-4A_{2}(A_{4}A_{6}^{2}+A_{3}A_{6}(A_{5}+A_{6})+A_{2}(A_{5}+A_{6})((2+A_{1})A_{6}+2A_{7})+A_{3}(2(1+A_{1})A_{6}+A_{7})A_{8}+A_{2}(2(1+A_{1})A_{6}+(2+A_{1})A_{7})A_{8})$, and $\gamma_{5}=4A_{2}(A_{6}+A_{1}A_{6}+A_{7})(A_{3}A_{6}+A_{2}(A_{6}+A_{7}))$.

Remark 3.2.1 In section 5, rigorous numerical analysis is used to demonstrate the existence of coexisting equilibrium point.

### 3.3 Local stability analysis

The linear approximation of the system (3) is considered around each equilibrium state in order to analyse the local behaviour of the system (3). The variational matrix of the linearized system at the point $\left(S,P_{1},P_{2}\right)$ is denoted by

$$J(S,P_{1},P_{2})=\left[\begin{array}{ccc} 1-2S-P_{1}-P_{2} & -S & -S\\ \frac{A_{4}P_{1}}{A_{1}+P_{2}} & \frac{A_{4}S}{A_{1}+P_{2}}-A_{2}P_{2}-A_{3} & -\frac{A_{4}SP_{1}}{{\left(A_{1}+P_{2}\right)}^{2}}-A_{2}P_{1}\\ A_{5}P_{2}+A_{8}P_{1}P_{2} & -P_{2}A_{6}+A_{8}P_{2}S & A_{5}S-P_{1}A_{6}-A_{7}+A_{8}P_{1}S \end{array}\right]$$

Let us assume, the characteristic equation of $J(S,P_{1},P_{2})$ at $E^{⁎}$ is given by

$$\lambda^{3}+G_{11}\lambda^{2}+G_{22}\lambda +G_{33}=0$$

Theorem 4 *The nature of the equilibrium points of the system* (3) *are as follows:*

(I) *The extinct equilibrium point* $E_{0}$ *always exhibits instability.*
(II) *The predator free equilibrium* $E_{1}$ *is considered to be locally asymptotically stable if the following criteria hold:*
  *(i)* $A_{1}A_{3}>A_{4}$ *and (ii)* $A_{7}>A_{5}$ *hold.*
(III) *The predator-free equilibrium point* $E_{2}$ *is locally asymptotically stable if certain requirements are satisfied:*
  *(i)* $A_{1}A_{3}<A_{4}$ *, (ii)* $A_{5}\leq \frac{A_{4}A_{6}-A_{1}A_{3}A_{6}}{A_{1}A_{3}}$ *and (iii)* $A_{8}<\frac{A_{1}A_{3}A_{4}A_{5}+A_{1}A_{3}A_{4}A_{6}-A_{4}^{2}A_{6}-A_{4}^{2}A_{7}}{A_{1}^{2}A_{3}^{2}-A_{1}A_{3}A_{4}}$ *hold.*
(IV) $E_{3}$ *is locally asymptotically stable if conditions*
  *(i)* $A_{5}>A_{7}$ *and (ii)* $A_{4}\leq \frac{A_{2}A_{5}^{2}-2A_{2}A_{5}A_{7}+A_{2}A_{7}^{2}+A_{3}A_{5}^{2}-A_{3}A_{5}A_{7}}{A_{5}A_{7}}$ *hold.*
(V) *Local asymptotic stability of the coexistence equilibrium state* $E^{⁎}$ *holds if and only if* $G_{11},G_{22},G_{33}$ *and* $G_{11}G_{22}-G_{33}$ *are positive, where* $G_{11},G_{22}$ *and* $G_{33}$ *have usual meanings.*

Proof i) The Jacobian matrix in the neighbourhood of the extinct equilibrium point $E_{0}$

$$J(E_{0})=\left[\begin{array}{ccc} 1 & 0 & 0\\ 0 & -A_{3} & 0\\ 0 & 0 & -A_{7} \end{array}\right]$$

The variational matrix $J(E_{0})$ has eigenvalues 1, -$A_{3}$, and -$A_{7}$. Here, 1>0, -$A_{3}<0$, and -$A_{7}<0$. Thus, the bio-system (3) is unstable near the extinct equilibrium point $E_{0}$.

ii) The Jacobian matrix around $E_{1}$ is

$$J(E_{1})=\left[\begin{array}{ccc} -1 & -1 & -1\\ 0 & \frac{A_{4}-A_{1}A_{3}}{A_{1}} & 0\\ 0 & 0 & A_{5}-A_{7} \end{array}\right]$$

Now, J($E_{1}$) has eigenvalues -1, $\frac{A_{4}-A_{1}A_{3}}{A_{1}}$, and $A_{5}-A_{7}$. Now, all three eigenvalues become negative if $A_{1}A_{3}>A_{4}$, and $A_{7}>A_{5}$ hold. If both the aforementioned requirements are fulfilled simultaneously, $E_{1}$ is locally asymptotically stable. If either $A_{1}A_{3}<A_{4}$ or $A_{7}<A_{5}$ or both are true, $E_{1}$ becomes unstable.

iii) The Jacobian matrix around $E_{2}$ is

$$J(E_{2})=\left[\begin{array}{ccc} x_{11} & x_{12} & x_{13}\\ x_{21} & x_{22} & x_{23}\\ x_{31} & x_{32} & x_{33} \end{array}\right]$$

where, $x_{11}=1-2B_{1}-c_{1}$, $x_{12}=-B_{1}$, $x_{13}=-B_{1}$, $x_{21}=\frac{A_{4}C_{1}}{A_{1}}$, $x_{22}=0$, $x_{23}=-\frac{A_{4}B_{1}C_{1}}{A_{1}^{2}}-A_{2}C_{1}$, and $x_{31}=0$, $x_{32}=0$, $x_{33}=A_{5}B_{1}-C_{1}A_{6}-A_{7}+A_{8}B_{1}C_{1}$.

The characteristic equation of J($E_{2}$) is

$$\kappa^{3}+F_{11}\kappa^{2}+F_{22}\kappa +F_{33}=0,$$

where, $F_{11}=-(x_{11}+x_{33})$, $F_{22}=x_{11}x_{33}-x_{11}x_{21}$ and $F_{33}=x_{12}x_{21}x_{33}$.

Taking, $A_{1}A_{3}<A_{4}$, $A_{5}>\frac{A_{4}A_{6}-A_{1}A_{3}A_{6}}{A_{1}A_{3}}$, $A_{8}<\frac{A_{1}A_{3}A_{4}A_{5}+A_{1}A_{3}A_{4}A_{6}-A_{4}^{2}A_{6}-A_{4}^{2}A_{7}}{A_{1}^{2}A_{3}^{2}-A_{1}A_{3}A_{4}}$, and $A_{7}>\frac{\left(A_{1}A_{3}A_{5}+A_{1}A_{3}A_{6}-A_{4}A_{6}\right)}{A_{4}}$, $F_{i}>0$ where i = 1, 2, 3 and $F_{11}F_{22}>F_{33}$. Hence, by Routh-Hurwitz criterion the system (3) is asymptotically stable in the neighbourhood of $E_{2}$.

iv) The Jacobian matrix around $E_{3}$ is given by

$$J(E_{3})=\left[\begin{array}{ccc} y_{11} & y_{12} & y_{13}\\ y_{21} & y_{22} & y_{23}\\ y_{31} & y_{32} & y_{33} \end{array}\right]$$

where, $y_{11}=1-2B_{2}-D_{1}$, $y_{12}=-B_{2}$, $y_{13}=-B_{2}$, $y_{21}=0$, $y_{22}=\frac{A_{4}B_{2}}{A_{1}+D_{1}}-A_{2}D_{1}-A_{3}$, $x_{23}=0$, $y_{31}=A_{5}D_{1}$, $y_{32}=A_{8}D_{1}B_{2}-A_{6}D_{1}$, and $y_{33}=0$.

The characteristic equation of J($E_{3}$) is

$$\Lambda^{3}+H_{11}\Lambda^{2}+H_{22}\Lambda +H_{33}=0,$$

where, $H_{11}=-(y_{11}+y_{22})$, $H_{22}=y_{11}y_{22}-y_{13}y_{31}$, and $H_{33}=y_{13}y_{31}y_{22}$.

Taking $A_{5}>A_{7}$ and $A_{4}\leq \frac{A_{2}A_{5}^{2}-2A_{2}A_{5}A_{7}+A_{2}A_{7}^{2}+A_{3}A_{5}^{2}-A_{3}A_{5}A_{7}}{A_{5}A_{7}}$, we get $H_{i}>0$ where i = 1, 2, 3 and $H_{11}H_{22}>H_{33}$. Hence, by Routh-Hurwitz criteria, the system (3) is asymptotically stable in the neighbourhood of $E_{3}$.

v) The Jacobian matrix near $E^{⁎}$ is given by

$$J(E^{⁎})=\left[\begin{array}{ccc} z_{11} & z_{12} & z_{13}\\ z_{21} & z_{22} & z_{23}\\ z_{31} & z_{32} & z_{33} \end{array}\right]$$

where, $z_{11}=1-2S^{⁎}-P_{1}^{⁎}-P_{2}^{⁎},z_{12}=-S^{⁎},z_{13}=-S^{⁎}$, $z_{21}=\frac{A_{4}P_{1}^{⁎}}{A_{1}+P_{2}^{⁎}},z_{22}=\frac{A_{4}S^{⁎}}{A_{1}+P_{2}^{⁎}}-A_{2}P_{2}^{⁎}-A_{3},z_{23}=-\frac{A_{4}S^{⁎}P_{1}^{⁎}}{{\left(A_{1}+P_{2}^{⁎}\right)}^{2}}-A_{2}P_{1}^{⁎}$, $z_{31}=A_{5}P_{2}^{⁎}+A_{8}P_{1}^{⁎}P_{2}^{⁎},z_{32}=-A_{6}P_{2}^{⁎}+A_{8}P_{2}^{⁎}S^{⁎}$, and $z_{33}=A_{5}S^{⁎}-P_{1}^{⁎}A_{6}-A_{7}+A_{8}P_{1}^{⁎}S^{⁎}$

The characteristic equation $J(E^{⁎})$ is

(7) $$\lambda^{3}+G_{11}\lambda^{2}+G_{22}\lambda +G_{33}=0$$

where, $G_{11}=-(z_{11}+z_{22}+z_{33})$, $G_{22}=(z_{11}z_{33}+z_{11}z_{22}+z_{22}z_{33}-z_{12}z_{21}-z_{13}z_{31}-z_{23}z_{32})$, and $G_{33}=-(z_{11}(z_{22}z_{33}-z_{23}z_{32})+z_{12}(z_{23}z_{31}-z_{21}z_{33})+z_{13}(z_{21}z_{32}-z_{22}z_{31}))$.

According to the Routh-Hurwitz criterion, the coexisting equilibrium $E^{⁎}$ is locally asymptotically stable if and only if the requirements $G_{11}>0$, $G_{33}>0$, and $G_{11}G_{22}-G_{33}>0$ are satisfied. □

The following Table 1 presents the local stability criteria for all ecologically viable equilibrium points of the system (3).

**Table 1:** Types of stability and stability conditions of all the ecologically feasible equilibrium states.

Equilibrium State	Stability Type	Stability condition
*E*_0_ = (0, 0, 0)	Unstable Saddle point	-
*E*_1_ = (1,0,0)	locally asymptotically stable	*A*_1_*A*_3_ > *A*_4_ and *A*_7_ > *A*_5_
*E*_2_ = (*B*_1_,*C*_1_,0)	locally asymptotically stable	*A*_1_*A*_3_ < *A*_4_, $A_{5}\leq \frac{A_{4}A_{6}-A_{1}A_{3}A_{6}}{A_{1}A_{3}}$, and $A_{8}<\frac{A_{1}A_{3}A_{4}A_{5}+A_{1}A_{3}A_{4}A_{6}-A_{4}^{2}A_{6}-A_{4}^{2}A_{7}}{A_{1}^{2}A_{3}^{2}-A_{1}A_{3}A_{4}}$
*E*_3_ = (*B*_2_,0,*D*_1_)	locally asymptotically stable	*A*_5_ > *A*_7_ and $A_{4}\leq \frac{A_{2}A_{5}^{2}-2A_{2}A_{5}A_{7}+A_{2}A_{7}^{2}+A_{3}A_{5}^{2}-A_{3}A_{5}A_{7}}{A_{5}A_{7}}$
*E*^⁎^=($S^{⁎},P_{1}^{⁎},P_{2}^{⁎}$)	locally asymptotically stable	*G*_11_ > 0,*G*_33_ > 0, and *G*_11_*G*_22_ − *G*_33_ > 0

Remark 3.3.1 Local stability around $E_{1}$ rules out the possibility of the existence of the equilibrium points $E_{2}$ and $E_{3}$.

### 3.4 Global stability

This section is dedicated to examining the global stability of both the axial and interior equilibria. Theorems related to this are provided as follows:

Theorem 5 *Global asymptotic stability of the axial equilibrium point*$E_{1}$*is ensured by the following sufficient conditions:*$A_{4}L_{2}\varrho_{2}\rho_{2}<A_{1}\Gamma_{1}(A_{2}L_{2}\varrho_{1}+A_{7}L_{3})$*and*$L_{1}(\varrho_{2}+\Gamma_{2})+L_{3}(A_{5}+A_{8}\varrho_{2})\Gamma_{2}\rho_{2}<A_{3}L_{2}\varrho_{1}$*.*

Proof For investigation of the globally asymptotic stability of the biosystem (3) around $E_{1}$, a positive definite Lyapunov function is taken into account:

$$V(S,P_{1},P_{2})=L_{1}(S-S^{⁎}-S^{⁎}ln\frac{S}{S^{⁎}})+L_{2}P_{1}+L_{3}P_{2}$$

Now taking derivative of $V(S,P_{1},P_{2})$ with respect to time, along the solutions of system (3), ˙$V(S,P_{1},P_{2})$ is given by,

$$\frac{dV(S,P_{1},P_{2})}{dt}=L_{1}\frac{\left(S-S^{⁎}\right)}{S}\frac{dS}{dt}+L_{2}\frac{dP_{1}}{dt}+L_{3}\frac{dP_{2}}{dt}$$

Let,

$$C_{1}=L_{1}\frac{\left(S-S^{⁎}\right)}{S}\frac{dS}{dt},C_{2}=L_{2}\frac{dP_{1}}{dt},C_{3}=L_{3}\frac{dP_{2}}{dt}$$

Now,

$$C_{1}\leq -L_{1}(P_{1}+P_{2})(S-1)$$

Let us consider $\rho_{1}<S<\rho_{2}$, $\varrho_{1}<P_{1}<\varrho_{2}$ and $\Omega_{1}<P_{2}<\Omega_{2}$ and then rearranging the terms

$$C_{1}+C_{2}+C_{3}\leq {ϒ}_{1}=\Omega_{1}+\Omega_{2}$$

where,

$${ϒ}_{1}=L_{1}P_{1}-A_{3}L_{2}P_{1}+L_{1}P_{2}-A_{7}L_{3}P_{2}-A_{2}L_{2}P_{1}P_{2}-A_{6}L_{3}P_{1}P_{2}-L_{1}P_{1}S-L_{1}P_{2}S+A_{5}L_{3}P_{2}S+A_{8}L_{3}P_{1}P_{2}S+\frac{A_{4}L_{2}P_{1}S}{A_{1}+P_{2}},\Omega_{1}=L_{1}P_{1}+L_{1}P_{2}+A_{5}L_{3}P_{2}S+A_{8}L_{3}P_{1}P_{2}S+\frac{A_{4}L_{2}P_{1}S}{A_{1}+P_{2}}\leq L_{1}P_{1}+L_{1}P_{2}+A_{5}L_{3}P_{2}S+A_{8}L_{3}P_{1}P_{2}S+\frac{A_{4}L_{2}P_{1}S}{A_{1}}\leq L_{1}\varrho_{2}+L_{1}\Gamma_{2}+A_{5}L_{3}\Gamma_{2}\rho_{2}+A_{8}L_{3}\varrho_{2}\Gamma_{2}\rho_{2}+\frac{A_{4}L_{2}\varrho_{2}\rho_{2}}{A_{1}},\Omega_{2}=-A_{3}L_{2}P_{1}-A_{7}L_{3}P_{2}-A_{2}L_{2}P_{1}P_{2}-A_{6}L_{3}P_{1}P_{2}-L_{1}P_{1}S-L_{1}P_{2}S\leq -A_{3}L_{2}\varrho_{1}-A_{7}L_{3}\Gamma_{1}-A_{2}L_{2}\varrho_{1}\Gamma_{1}-A_{6}L_{3}\varrho_{1}\Gamma_{1}-L_{1}\varrho_{1}\rho_{1}-L_{1}\Gamma_{1}\rho_{1}$$

So, $C_{1}+C_{2}+C_{3}\leq L_{1}\varrho_{2}+L_{1}\Gamma_{2}+A_{5}L_{3}\Gamma_{2}\rho_{2}+A_{8}L_{3}\varrho_{2}\Gamma_{2}\rho_{2}+\frac{A_{4}L_{2}\varrho_{2}\rho_{2}}{A_{1}}-A_{3}L_{2}\varrho_{1}-A_{7}L_{3}\Gamma_{1}-A_{2}L_{2}\varrho_{1}\Gamma_{1}-A_{6}L_{3}\varrho_{1}\Gamma_{1}-L_{1}\varrho_{1}\rho_{1}-L_{1}\Gamma_{1}\rho_{1}<0$; provided, $A_{4}L_{2}\varrho_{2}\rho_{2}<A_{1}\Gamma_{1}(A_{2}L_{2}\varrho_{1}+A_{7}L_{3})$ and $L_{1}(\varrho_{2}+\Gamma_{2})+L_{3}(A_{5}+A_{8}\varrho_{2})\Gamma_{2}\rho_{2}<A_{3}L_{2}\varrho_{1}$.

Thus, $\frac{dV}{dt}<0$ under the conditions: $A_{4}L_{2}\varrho_{2}\rho_{2}<A_{1}\Gamma_{1}(A_{2}L_{2}\varrho_{1}+A_{7}L_{3})$ and $L_{1}(\varrho_{2}+\Gamma_{2})+L_{3}(A_{5}+A_{8}\varrho_{2})\Gamma_{2}\rho_{2}<A_{3}L_{2}\varrho_{1}$. □

Theorem 6 *The equilibrium point* $E^{⁎}$ *is proven to be globally asymptotically stable given the specified conditions* $P_{2}^{⁎}\leq \Theta_{2}$ *,* $L_{2}>{ϒ}_{2}$ *,* $A_{5}\vartheta_{1}(P_{1}^{⁎}P_{2}^{⁎}(A_{6}-A_{8}S^{⁎})+A_{5}(\vartheta_{1}\theta_{1}-S^{⁎}\vartheta_{2}))>0$ *,* $A_{4}<{ϒ}_{3}$ *, and* $\vartheta_{1}>{ϒ}_{4}$ *hold.*

Proof For investigation of the globally asymptotic stability of the biosystem (3) around the coexisting equilibrium $E^{⁎}$, a positive definite Lyapunov function is taken into account:

$$W(S,P_{1},P_{2})=L_{1}(S-S^{⁎}-S^{⁎}ln\frac{S}{S^{⁎}})+L_{2}(P_{1}-P_{1}^{⁎}-P_{1}^{⁎}ln\frac{P_{1}}{P_{1}^{⁎}})+L_{3}(P_{2}-P_{2}^{⁎}-P_{2}^{⁎}ln\frac{P_{2}}{p_{2}^{⁎}})$$

Now taking the time derivative of $W(S,P_{1},P_{2})$ along the solutions of system (3), we get

$$\frac{dW(S,P_{1},P_{2})}{dt}=L_{1}\frac{\left(S-S^{⁎}\right)}{S}\frac{dS}{dt}+L_{2}\frac{\left(P_{1}-P_{1}^{⁎}\right)}{P_{1}}\frac{dP_{1}}{dt}+L_{3}\frac{\left(P_{2}-P_{2}^{⁎}\right)}{P_{2}}\frac{dP_{2}}{dt}$$

Let, $D_{1}=L_{1}\frac{\left(S-S^{⁎}\right)}{S}\frac{dS}{dt}$, $D_{2}=L_{2}\frac{\left(P_{1}-P_{1}^{⁎}\right)}{P_{1}}\frac{dP_{1}}{dt}$, $D_{3}=L_{3}\frac{\left(P_{2}-P_{2}^{⁎}\right)}{P_{2}}\frac{dP_{2}}{dt}$

Now, $D_{1}\leq -L_{1}(P_{1}-P_{1}^{⁎}+P_{2}-P_{2}^{⁎})(S-S^{⁎})$, $D_{2}=L_{2}(P_{1}-P_{1}^{⁎})(A_{2}(-P_{2}+P_{2}^{⁎})+\frac{\left(A_{4}(P_{2}^{⁎}S+A_{1}(S-S^{⁎})-P_{2}S^{⁎})\right)}{\left((A_{1}+P_{2})(A_{1}+P_{2}^{⁎})\right)})$, and $D_{3}=-L_{3}(P_{2}-P_{2}^{⁎})(A_{6}(P_{1}-P_{1}^{⁎})-A_{8}P_{1}S+A_{8}P_{1}^{⁎}S^{⁎}+A_{5}(-s+S^{⁎}))$.

Taking $L_{1}=A_{5}L_{3}$,

$$D_{1}+D_{2}+D_{3}\leq -A_{2}L_{2}P_{1}P_{2}-A_{6}L_{3}P_{1}P_{2}+A_{2}L_{2}P_{1}^{⁎}P_{2}+A_{6}L_{3}P_{1}^{⁎}P_{2}+A_{2}L_{2}P_{1}P_{2}^{⁎}+A_{6}L_{3}P_{1}P_{2}^{⁎}-A_{2}L_{2}P_{1}^{⁎}P_{2}^{⁎}-A_{6}L_{3}P_{1}^{⁎}P_{2}^{⁎}-A_{5}L_{3}P_{1}S+A_{5}L_{3}P_{1}^{⁎}S+A_{8}L_{3}P_{1}P_{2}S-A_{8}L_{3}P_{1}P_{2}^{⁎}S+A_{5}L_{3}P_{1}S^{⁎}++\upsilon_{1}+\upsilon_{2}-A_{5}L_{3}P_{1}^{⁎}S^{⁎}-A_{8}L_{3}P_{1}^{⁎}P_{2}S^{⁎}+A_{8}L_{3}P_{1}^{⁎}P_{2}^{⁎}S^{⁎}\leq -A_{2}L_{2}P_{1}P_{2}-A_{6}L_{3}P_{1}P_{2}+A_{2}L_{2}P_{1}^{⁎}P_{2}+A_{6}L_{3}P_{1}^{⁎}P_{2}+A_{2}L_{2}P_{1}P_{2}^{⁎}+A_{6}L_{3}P_{1}P_{2}^{⁎}-A_{2}L_{2}P_{1}^{⁎}P_{2}^{⁎}-A_{6}L_{3}P_{1}^{⁎}P_{2}^{⁎}-A_{5}L_{3}P_{1}S+A_{5}L_{3}P_{1}^{⁎}S+A_{8}L_{3}P_{1}P_{2}S-A_{8}L_{3}P_{1}P_{2}^{⁎}S+A_{5}L_{3}P_{1}S^{⁎}+\upsilon_{3}+\upsilon_{4}-A_{5}L_{3}P_{1}^{⁎}S^{⁎}-A_{8}L_{3}P_{1}^{⁎}P_{2}S^{⁎}+A_{8}L_{3}P_{1}^{⁎}P_{2}^{⁎}S^{⁎}$$

Now, let us consider $\theta_{1}<S<\theta_{2},\vartheta_{1}<P_{1}<\vartheta_{2}$, and $\Theta_{1}<P_{2}<\Theta_{2}$, then rearranging the terms

$$D_{1}+D_{2}+D_{3}\leq \delta_{1}+\delta_{2}$$

where,

$$\delta_{1}=A_{2}L_{2}P_{1}^{⁎}P_{2}+A_{6}L_{3}P_{1}^{⁎}P_{2}+A_{2}L_{2}P_{1}P_{2}^{⁎}+A_{6}L_{3}P_{1}P_{2}^{⁎}+A_{5}L_{3}P_{1}^{⁎}S+A_{8}L_{3}P_{1}P_{2}S+\upsilon_{5}+A_{5}L_{3}P_{1}S^{⁎}+A_{8}L_{3}P_{1}^{⁎}P_{2}^{⁎}S^{⁎}\leq A_{2}L_{2}P_{1}^{⁎}\Theta_{2}+A_{6}L_{3}P_{1}^{⁎}\Theta_{2}+A_{2}L_{2}\vartheta_{2}P_{2}^{⁎}+A_{6}L_{3}\vartheta_{2}P_{2}^{⁎}+A_{5}L_{3}P_{1}^{⁎}\theta_{2}+A_{8}L_{3}\vartheta_{2}\Theta_{2}\theta_{2}+\upsilon_{6}+A_{5}L_{3}\vartheta_{2}S^{⁎}+A_{8}L_{3}P_{1}^{⁎}P_{2}^{⁎}S^{⁎}=\delta_{1}^{\prime },\delta_{2}=-A_{2}L_{2}P_{1}P_{2}-A_{6}L_{3}P_{1}P_{2}-A_{2}L_{2}P_{1}^{⁎}P_{2}^{⁎}-A_{6}L_{3}P_{1}^{⁎}P_{2}^{⁎}-A_{5}L_{3}P_{1}S-A_{8}L_{3}P_{1}P_{2}^{⁎}S+\upsilon_{7}-A_{5}L_{3}P_{1}^{⁎}S^{⁎}-A_{8}L_{3}P_{1}^{⁎}P_{2}S^{⁎}\leq -A_{2}L_{2}\vartheta_{1}\Theta_{1}-A_{6}L_{3}\vartheta_{1}\Theta_{1}-A_{2}L_{2}P_{1}^{⁎}P_{2}^{⁎}-A_{6}L_{3}P_{1}^{⁎}P_{2}^{⁎}-A_{5}L_{3}\vartheta_{1}\theta_{1}-A_{8}L_{3}\vartheta_{1}P_{2}^{⁎}\theta_{1}+\upsilon_{8}-A_{5}L_{3}P_{1}^{⁎}S^{⁎}-A_{8}L_{3}P_{1}^{⁎}\Theta_{1}S^{⁎}=\delta_{2}^{\prime }$$

and, $\upsilon_{1}=\frac{\left(A_{1}A_{4}L_{2}P_{1}S\right)}{\left(A_{1}+P_{2})(A_{1}+P_{2}^{⁎}\right)}-\frac{\left(A_{1}A_{4}L_{2}P_{1}^{⁎}S\right)}{\left(A_{1}+P_{2})(A_{1}+P_{2}^{⁎}\right)}+\frac{\left(A_{4}L_{2}P_{1}P_{2}^{⁎}S\right)}{\left(A_{1}+P_{2})(A_{1}+P_{2}^{⁎}\right)}-\frac{\left(A_{4}L_{2}P_{1}^{⁎}P_{2}^{⁎}S\right)}{\left(A_{1}+P_{2})(A_{1}+P_{2}^{⁎}\right)}$, $\upsilon_{2}=-\frac{\left(A_{1}A_{4}L_{2}P_{1}S^{⁎}\right)}{\left(A_{1}+P_{2})(A_{1}+P_{2}^{⁎}\right)}+\frac{\left(A_{1}A_{4}L_{2}P_{1}^{⁎}S^{⁎}\right)}{\left(A_{1}+P_{2})(A_{1}+P_{2}^{⁎}\right)}-\frac{\left(A_{4}L_{2}P_{1}P_{2}S^{⁎}\right)}{\left(A_{1}+P_{2})(A_{1}+P_{2}^{⁎}\right)}+\frac{\left(A_{4}L_{2}P_{1}^{⁎}P_{2}S^{⁎}\right)}{\left(A_{1}+P_{2})(A_{1}+P_{2}^{⁎}\right)}$, $\upsilon_{3}=\frac{\left(A_{1}A_{4}L_{2}P_{1}S\right)}{\left(A_{1})(A_{1}+P_{2}^{⁎}\right)}-\frac{\left(A_{1}A_{4}L_{2}P_{1}^{⁎}S\right)}{\left(A_{1}+P_{2})(A_{1}+P_{2}^{⁎}\right)}+\frac{\left(A_{4}L_{2}P_{1}P_{2}^{⁎}S\right)}{\left(A_{1})(A_{1}+P_{2}^{⁎}\right)}-\frac{\left(A_{4}L_{2}P_{1}^{⁎}P_{2}^{⁎}S\right)}{\left(A_{1}+P_{2})(A_{1}+P_{2}^{⁎}\right)}$, $\upsilon_{4}=-\frac{\left(A_{1}A_{4}L_{2}P_{1}S^{⁎}\right)}{\left(A_{1}+P_{2})(A_{1}+P_{2}^{⁎}\right)}+\frac{\left(A_{1}A_{4}L_{2}P_{1}^{⁎}S^{⁎}\right)}{\left(A_{1})(A_{1}+P_{2}^{⁎}\right)}-\frac{\left(A_{4}L_{2}P_{1}P_{2}S^{⁎}\right)}{\left(A_{1}+P_{2})(A_{1}+P_{2}^{⁎}\right)}+\frac{\left(A_{4}L_{2}P_{1}^{⁎}P_{2}S^{⁎}\right)}{\left(P_{2})(A_{1}+P_{2}^{⁎}\right)}$, $\upsilon_{5}=\frac{\left(A_{1}A_{4}L_{2}P_{1}S\right)}{\left(A_{1})(A_{1}+P_{2}^{⁎}\right)}+\frac{\left(A_{4}L_{2}P_{1}P_{2}^{⁎}S\right)}{\left(A_{1})(A_{1}+P_{2}^{⁎}\right)}+\frac{\left(A_{1}A_{4}L_{2}P_{1}^{⁎}S^{⁎}\right)}{\left(A_{1})(A_{1}+P_{2}^{⁎}\right)}+\frac{\left(A_{4}L_{2}P_{1}^{⁎}P_{2}S^{⁎}\right)}{\left(P_{2})(A_{1}+P_{2}^{⁎}\right)}$, $\upsilon_{6}=\frac{\left(A_{1}A_{4}L_{2}\vartheta_{2}\theta_{2}\right)}{\left(A_{1})(A_{1}+P_{2}^{⁎}\right)}+\frac{\left(A_{4}L_{2}\vartheta_{2}P_{2}^{⁎}\theta_{2}\right)}{\left(A_{1})(A_{1}+P_{2}^{⁎}\right)}+\frac{\left(A_{1}A_{4}L_{2}P_{1}^{⁎}S^{⁎}\right)}{\left(A_{1})(A_{1}+P_{2}^{⁎}\right)}+\frac{\left(A_{4}L_{2}P_{1}^{⁎}\Theta_{2}S^{⁎}\right)}{\left(\Theta_{2})(A_{1}+P_{2}^{⁎}\right)}$, $\upsilon_{7}=-\frac{\left(A_{1}A_{4}L_{2}P_{1}^{⁎}S\right)}{\left(A_{1}+P_{2})(A_{1}+P_{2}^{⁎}\right)}-\frac{\left(A_{4}L_{2}P_{1}^{⁎}P_{2}^{⁎}S\right)}{\left(A_{1}+P_{2})(A_{1}+P_{2}^{⁎}\right)}-\frac{\left(A_{1}A_{4}L_{2}P_{1}S^{⁎}\right)}{\left(A_{1}+P_{2})(A_{1}+P_{2}^{⁎}\right)}-\frac{\left(A_{4}L_{2}P_{1}P_{2}S^{⁎}\right)}{\left(A_{1}+P_{2})(A_{1}+P_{2}^{⁎}\right)}$, and $\upsilon_{8}=-\frac{\left(A_{1}A_{4}L_{2}P_{1}^{⁎}\theta_{1}\right)}{\left(A_{1}+\Theta_{1})(A_{1}+P_{2}^{⁎}\right)}-\frac{\left(A_{4}L_{2}P_{1}^{⁎}P_{2}^{⁎}\theta_{1}\right)}{\left(A_{1}+\Theta_{1})(A_{1}+P_{2}^{⁎}\right)}-\frac{\left(A_{1}A_{4}L_{2}\vartheta_{1}S^{⁎}\right)}{\left(A_{1}+\Theta_{1})(A_{1}+P_{2}^{⁎}\right)}-\frac{\left(A_{4}L_{2}\vartheta_{1}\Theta_{1}S^{⁎}\right)}{\left(A_{1}+\Theta_{1})(A_{1}+P_{2}^{⁎}\right)}$.

Now, $D_{1}+D_{2}+D_{3}\leq \delta_{1}^{\prime }+\delta_{2}^{\prime }<0$, provided

$L_{2}>\frac{\left(L_{3}(A_{6}P_{2}^{⁎}\vartheta_{2}+A_{6}P_{1}^{⁎}\Theta_{2}+A_{5}P_{1}^{⁎}\theta_{2}+A_{8}\vartheta_{2}\Theta_{2}\theta_{2})\right)}{A_{2}(\vartheta_{1}\Theta_{1}-P_{2}^{⁎}\vartheta_{2}+P_{1}^{⁎}(P_{2}^{⁎}-\Theta_{2}))}={ϒ}_{2}$, $A_{5}\vartheta_{1}(P_{1}^{⁎}P_{2}^{⁎}(A_{6}-A_{8}S^{⁎})+A_{5}(\vartheta_{1}\theta_{1}-S^{⁎}\vartheta_{2}))>0$,

$A_{4}<\frac{\left(A_{1}A_{6}\vartheta_{1}\Theta_{1}L_{3}(A_{1}+P_{2}^{⁎})\right)}{L_{2}(2A_{1}P_{1}^{⁎}S^{⁎}+A_{1}\vartheta_{2}\theta_{2}+P_{2}^{⁎}\vartheta_{2}\theta_{2})}={ϒ}_{3}$, $\vartheta_{1}>\frac{\left(P_{2}^{⁎}\vartheta_{2}+P_{1}^{⁎}(-P_{2}^{⁎}+\Theta_{2})\right)}{\Theta_{1}}={ϒ}_{4}$, and $P_{2}^{⁎}\leq \Theta_{2}$

Thus, $\frac{dW}{dt}<0$ under the conditions: $L_{2}>{ϒ}_{2}$, $A_{5}\vartheta_{1}(P_{1}^{⁎}P_{2}^{⁎}(A_{6}-A_{8}S^{⁎})+A_{5}(\vartheta_{1}\theta_{1}-S^{⁎}\vartheta_{2}))>0$, $A_{4}<{ϒ}_{3}$, $\vartheta_{1}>{ϒ}_{4}$, and $P_{2}^{⁎}\leq \Theta_{2}$. □

## 4 Qualitative changes in the ecological scenario

In general, fixed points' parametric region describing global asymptotic stability and any parametric region describing Hopf bifurcation or transcritical bifurcation intersect at their boundaries. This is due to the qualitative nature of the two qualitative scenarios, the attractor's basin of attraction and its bifurcation. Stability or the direction of stability may change in an ecosystem once the influencing parameters are varied. Bifurcations within an ecological system hold significance due to their potential to instigate the emergence of novel behaviours within said system. We theoretically discuss some of the bifurcations of codimension one in this section.

### 4.1 Transcritical bifurcation

The phenomenon of transcritical bifurcation holds considerable significance within the realm of dynamical systems and nonlinear dynamics. It is a phenomenon that arises when a parameter traverses a critical value, leading to a change in the stability of an equilibrium point. Theorems relevant to this are provided.

Theorem 7 *(i) The system*(3)*exhibits a transcritical bifurcation as the fixed point*$E_{1}$*crosses the threshold value*$A_{5}=A_{7}$*provided*$A_{5}\neq 0$*.*

*(ii) The system*(3)*encounters a transcritical bifurcation near the fixed point*$E_{2}$*along the parametric surface*$A_{1}A_{3}A_{4}(A_{5}+A_{6}+A_{8})-A_{4}^{2}(A_{6}+A_{7})-A_{1}^{2}A_{3}^{2}A_{8}=0$*.*

*(iii) The system*(3)*confronts a transcritical bifurcation along the parametric surface*$-A_{2}A_{5}^{2}-A_{1}A_{2}A_{5}^{2}-A_{3}A_{5}^{2}-A_{1}A_{3}A_{5}^{2}+2A_{2}A_{5}A_{7}+A_{1}A_{2}A_{5}A_{7}+A_{3}A_{5}A_{7}+A_{4}A_{5}A_{7}-A_{2}A_{7}^{2}=0$*around the equilibrium point*$E_{3}$*.*

Proof (i) At $A_{5}=A_{7}=A_{5}^{tb}$. The system (3) encounters a transcritical bifurcation near the equilibrium point $E_{1}$ as $J(E_{1})$ has one eigenvalue zero. The Jacobian matrix at the equilibrium point $E_{1}$ at $A_{5}=A_{5}^{tb}$ is

$$J(E_{1})=\left[\begin{array}{ccc} -1 & -1 & -1\\ 0 & \frac{A_{4}-A_{1}A_{3}}{A_{1}} & 0\\ 0 & 0 & 0 \end{array}\right]$$

Now, $P={\left(p_{1},p_{2},p_{3}\right)}^{t}={\left(-1,0,1\right)}^{t}$, and $Q={\left(q_{1},q_{2},q_{3}\right)}^{t}={\left(0,0,1\right)}^{t}$ are two eigenvectors corresponding to the zero eigenvalue of the matrices $J(E_{1})$ and $J{\left(E_{1}\right)}^{T}$ respectively. After some calculation, we get

$$K_{A_{5}}(E_{1};A_{5}^{tb})=\left[\begin{array}{c} 0\\ 0\\ 0 \end{array}\right],D(K_{A_{5}}(E_{1};A_{5}^{tb}))P=\left[\begin{array}{ccc} 0 & 0 & 0\\ 0 & 0 & 0\\ 0 & 0 & 1 \end{array}\right]\left[\begin{array}{c} -1\\ 0\\ 1 \end{array}\right]=\left[\begin{array}{c} 0\\ 0\\ 1 \end{array}\right],\;\text{and}\;D^{2}(K_{A_{5}}(E_{1};A_{5}^{tb}))(P,P)=\left[\begin{array}{c} 0\\ 0\\ -A_{5} \end{array}\right].$$

Therefore, $Q^{T}(K_{A_{5}}(E_{1};A_{5}^{tb}))=0,Q^{T}(D(K_{A_{5}}(E_{1};A_{5}^{tb}))P)=1\neq 0$, and $Q^{T}(D^{2}(K_{A_{5}}(E_{1};A_{5}^{tb}))(P,P))=-A_{5}\neq 0$.

Therefore, a transcritical bifurcation occurs at $A_{5}=A_{5}^{tb}$ around the predator free equilibrium $E_{1}$. The proofs for (ii) and (iii) can be derived in a similar manner to that of (i). □

### 4.2 Hopf bifurcation

The Hopf bifurcation is a fundamental concept within the wider domain of bifurcation theory. It offers significant insights into the shift from stable behaviour to oscillatory behaviour. It serves as a crucial foundation for investigating more intricate bifurcation scenarios. Below are the theorems pertaining to Hopf bifurcation.

Theorem 8 *The system*(3)*does not experience Hopf bifurcation for any parameter at the equilibrium points*$E_{2}$*and*$E_{3}$*.*

Proof According to Liu [59], Hopf bifurcation around $E_{2}$ for a parameter value say $\mu =\mu_{0}$ exists iff conditions:

(i)$F_{i}(\mu_{0})>0$ for i=1,2,3 and $(V)(\mu_{0})=0$, where $V=F_{11}F_{22}-F_{33}$.

(ii) $\frac{dV}{d\mu }(\mu_{0})\neq 0$ both hold.

Solving V=0, we get-

$A_{7}=\frac{-2A_{1}^{2}A_{3}^{2}A_{8}+A_{1}A_{3}A_{4}(2A_{5}+2A_{6}+2A_{8}-1)-Z_{1}}{2A_{4}^{2}}$ and $A_{7}=\frac{-2A_{1}^{2}A_{3}^{2}A_{8}+A_{1}A_{3}A_{4}(2A_{5}+2A_{6}+2A_{8}-1)+Z_{1}}{2A_{4}^{2}}$

where, $Z_{1}=A_{4}(\sqrt{A_{3}(A_{1}^{2}A_{3}+4A_{1}A_{3}A_{4}-4A_{4}^{2})}+2A_{4}A_{6})$

putting first value of $A_{7}$ in $F_{11}$, it is found that

$F_{11}>0\Leftrightarrow \frac{4{A_{4}}^{2}}{{A_{1}}^{2}+4A_{1}A_{4}}\leq A_{3}<\frac{A_{4}}{A_{1}}$ and $F_{22}<0\Leftrightarrow A_{3}\geq \frac{4A_{4}^{2}}{A_{1}^{2}+4A_{1}A_{4}}$ Hence, $F_{11}$, $F_{22}$ cannot be positive simultaneously and hence the result.

Similarly, we can show that conditions (I) or (II) or both do not hold for the latter value of $A_{7}$, which implies the fact that the system (3) does not experience Hopf bifurcation around the equilibrium point $E_{2}$.

In a similar way, it can be shown that the system (3) does not experience Hopf bifurcation around the equilibrium point $E_{3}$ as well. □

Theorem 9 *The system*(3)*undergoes Hopf bifurcation around the equilibrium point*$E^{⁎}$*when*$A_{4}$*passes through the critical value*$A_{4}^{H⁎}$*where*$A_{4}^{H⁎}$*satisfies G(*$A_{4}^{H⁎}$*)=*$G_{11}(A_{4}^{H⁎})$$G_{22}(A_{4}^{H⁎})$*-*$G_{33}(A_{4}^{H⁎})$*=0,*$G_{i}(A_{4}^{H⁎})>0$*for i=1,2,3 and*$G_{11}(A_{4}^{H⁎})$$G_{22}^{\prime }$*(*$A_{4}^{H⁎}$*) +*$G_{22}(A_{4}^{H⁎})$$G_{11}^{\prime }$*(*$A_{4}^{H⁎}$*) -*$G_{33}^{\prime }$*(*$A_{4}^{H⁎}$*)* ≠0*. Where* $G_{11},G_{22}$ *and* $G_{33}$ *have their usual meanings.*

Proof By the condition G$(A_{4})=0$ at $A_{4}=A_{4}^{H⁎}$. The Characteristic equation (6) can be written as

(8) $$(\lambda^{2}+G_{22})(\lambda +G_{11})=0$$

Thus, solving (8) we have, $\lambda =-G_{11}$, ± $\sqrt{G_{22}}$ i; provided $G_{11}$, $G_{22}>0$. For $A_{4}$ ∈ ($A_{4}^{H⁎}$-*ϵ*, $A_{4}^{H⁎}$+*ϵ*), the general form of the roots are $\lambda_{1}=\zeta_{1}(A_{4})+\zeta_{2}(A_{4})$, $\lambda_{2}=\zeta_{1}(A_{4})-\zeta_{2}(A_{4})$ and $\lambda_{3}=-G_{11}(A_{4})$ Now, substituting $\lambda_{1}=\zeta_{1}(A_{4}^{H⁎})+\zeta_{2}(A_{4}^{H⁎})$ in equation (7) and then differentiating and separating the real and imaginary part, we get

(9) $$P(A_{4}){\zeta_{1}}^{\prime }(A_{4})-Q(A_{4})\zeta_{2}^{\prime }(A_{4})+R(A_{4})=0\;Q(A_{4})\zeta_{1}^{\prime }(A_{4})+P(A_{4})\zeta_{2}^{\prime }(A_{4})+S(A_{4})=0$$

where, $P(A_{4})=3\zeta_{1}^{2}-3\zeta_{2}^{2}+G_{22}+2G_{11}\zeta_{1}$, $Q(A_{4})=6\zeta_{1}\zeta_{2}+2G_{11}\zeta_{2}$, and $R(A_{4})=G_{11}^{\prime }\zeta_{1}^{2}-G_{11}^{\prime }\zeta_{2}^{2}+G_{22}^{\prime }\zeta_{1}+G_{33}^{\prime }$, $S(A_{4})=G_{22}^{\prime }\zeta_{2}+2\zeta_{1}\zeta_{2}$. From System (9), we can show that-

$${\zeta_{1}}^{\prime }(A_{4})=-\frac{P(A_{4})R(A_{4})+Q(A_{4})S(A_{4})}{P^{2}(A_{4})+Q^{2}(A_{4})}$$

Using $\zeta_{1}^{\prime }(A_{4}^{H⁎})=0$, $\zeta_{2}^{\prime }(A_{4}^{H⁎})=i\sqrt{G_{22}(A_{4}^{H⁎})}$, we get- $P(A_{4}^{H⁎})=-2G_{22}$, $Q(A_{4}^{H⁎})=2G_{11}\sqrt{G_{22}}$, and $R(A_{4}^{H⁎})=G_{33}^{\prime }-G_{11}^{\prime }G_{22}$, $S(A_{4}^{H⁎})=\sqrt{G_{22}}G_{22}^{\prime }$ Now, for Transversality condition, we need to show that-

$$\frac{d}{dA_{4}}{\left(Re(\lambda_{i}(A_{4}))\right)}_{A_{4}=A_{4}^{H⁎}}\neq 0;i=1,2,3$$

Hence, the following equation is obtained

$$\frac{d}{dA_{4}}{\left(Re(\lambda_{i}(A_{4}))\right)}_{A_{4}=A_{4}^{H⁎}}=-\frac{P(A_{4})R(A_{4})+Q(A_{4})S(A_{4})}{P^{2}(A_{4})+Q^{2}(A_{4})}=\frac{-2(G_{33}^{\prime }-G_{11}^{\prime }G_{22})+2G_{11}G_{22}G_{22}^{\prime }}{4(G_{22}^{2}+G_{11}^{2}G_{22})}=\frac{G_{11}^{\prime }G_{22}-G_{33}^{\prime }+G_{11}{G_{22}}^{\prime }}{2(G_{22}+G_{11}^{2})}$$

Now, if $G_{11}^{\prime }G_{22}-G_{33}^{\prime }+G_{11}{G_{22}}^{\prime }\neq 0$, then the transversality condition is satisfied, and hence the abovementioned system (3) experiences Hopf-bifurcation around the equilibrium point $E^{⁎}$ when $A_{4}$ passes through the critical value $A_{4}=A_{4}^{H⁎}$. The presence of Hopf bifurcation for other parameters can be demonstrated in a similar manner. Discussion pertaining to the direction and stability of Hopf bifurcations for different parameters is done numerically in Section 5.2.1. □

#### 4.2.1 Direction and stability of Hopf bifurcations around the interior equilibrium point $E^{⁎}$

This section presents a theorem that pertains to the direction and stability of Hopf bifurcations. Determining the direction of a Hopf bifurcation involves ascertaining the local existence of the bifurcating branch of periodic solutions for values of the bifurcating parameter that are either greater than or less than the critical point. On the other hand, the investigation of the stability of a Hopf bifurcation encompasses the examination of the system's behaviour and resilience during the transition from a stable equilibrium state to the occurrence of sustained oscillations.

Theorem 10 *[60]**The direction of the Hopf bifurcation and stability of the bifurcating periodic solution is determined by the sign of*$l_{1}$*at the hopf bifurcation point. Where,*$l_{1}$*is the first Lyapunov coefficient.*

1. *When the sign of*$l_{1}$*is positive, then the nature of the Hopf bifurcation is subcritical and the resulting bifurcating periodic solutions are unstable.*
2. *the sign of*$l_{1}$*is negative, then the nature of the Hopf bifurcation is supercritical and the resulting bifurcating periodic solutions are stable.*

Further, a necessary condition for the occurrence of a generalized Hopf bifurcation is that the first Lyapunov coefficient vanishes, i.e., $l_{1}=0$ and $l_{2}\neq 0$. Let the system undergo Hopf bifurcation at the parametric space value $\left(a_{1},a_{2},a_{3},a_{4},a_{5},a_{6},a_{7},a_{8}\right)$, and let (α,β,γ) be the corresponding fixed point. Let *A* be the Jacobian of the system at (α,β,γ). Let $q=(\eta_{1},\eta_{2},\eta_{3})$ be the eigen vector of *A* corresponding to the eigen value λ=iω,ω>0 and $\overset{¯}{q}=({\overset{¯}{\eta }}_{1},{\overset{¯}{\eta }}_{2},{\overset{¯}{\eta }}_{3})$. If $\overset{¯}{p}=({\overset{¯}{\mu }}_{1},{\overset{¯}{\mu }}_{2},{\overset{¯}{\mu }}_{3})$ is the eigen vector of $A^{T}$ corresponding to $\overset{¯}{\lambda }$, then $l_{1}$ as well as $l_{2}$ can be easily derived following the procedure from [60], [61].

## 5 Numerical simulations

Throughout our analysis, we have identified three crucial equilibrium points: $E_{2}$ (second predator free) and $E_{3}$ (first predator free), as well as the coexistence equilibrium point $E^{⁎}$. Here, we conduct numerical simulations of system (3) to demonstrate the results obtained from our theoretical analysis with the help of some software such as MATHEMATICA, MATLAB, and MATCONT [62]. Some ecologically possible parametric values have been considered, as illustrated in the table below:

**Table 2:** Parameter values for the system (3).

Parameter	value
*A*_1_	0.02
*A*_2_	0.05
*A*_3_	2
*A*_4_	0.4
*A*_5_	4.536
*A*_6_	0.052
*A*_7_	4.546
*A*_8_	114.98

### 5.1 Stability of the equilibrium points

In this section, at first, we compute and confirm numerically the parametric requirements for the presence and stability of all biologically possible equilibrium points.

**Figure 1:**
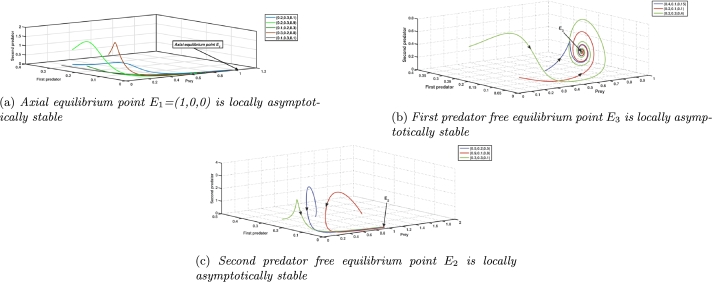
Local Stability of axial and boundary equilibrium points.

Taking parameter values as $A_{3}=0.875$, $A_{4}=0.0125$, $A_{5}=4.536$ and the rest values from Table 2, the stability conditions for local stability of the axial equilibrium point $E_{1}$ are satisfied as $A_{1}A_{3}=0.0175>A_{4}$ and $A_{7}=4.546>A_{5}$, which can be seen from the Fig. 1a. Various coextant initial populations lead to a predator-free equilibrium point through different trajectories, as shown in Fig. 1a, interpreting the fact that when two predator species go extinct, prey grows without any disturbances and eventually settles at the carrying capacity of the system, which is found to be true in nature.

To verify the stability conditions of the first predator free equilibrium point $E_{3}$, we consider $A_{3}=2.723732$, $A_{4}=0.516658$, $A_{5}=9.3241$ and the rest parameter values from Table 2, all the stability conditions $A_{5}=9.3241>A_{7}$ and $\frac{A_{2}A_{5}^{2}-2A_{2}A_{5}A_{7}+A_{2}A_{7}^{2}+A_{3}A_{5}^{2}-A_{3}A_{5}A_{7}}{A_{5}A_{7}}=2.88978>A_{4}$ are satisfied for these parameter values. It can be seen in Fig. 1b confirming the local stability of the first predator free equilibrium point $E_{3}$. From a biological perspective, when environmental parameters reach the specified values quantitatively, the coexisting population faces challenges in maintaining population mass balance, ultimately resulting in the extinction of the first predator in the ecosystem.

We consider $A_{3}=0.51558$, $A_{4}=0.010528$, $A_{5}=1.135458$, and the rest parameters from Table 2 to validate the stability conditions of the second predator free equilibrium point $E_{2}$. These parameter values satisfy all the stability conditions as $A_{1}A_{3}=0.0103116<A_{4}$, $\frac{A_{4}A_{6}-A_{1}A_{3}A_{6}}{A_{1}A_{3}}=0.00109531<A_{5}$, $\frac{A_{1}A_{3}A_{4}A_{5}+A_{1}A_{3}A_{4}A_{6}-A_{4}^{2}A_{6}-A_{4}^{2}A_{7}}{A_{1}^{2}A_{3}^{2}-A_{1}A_{3}A_{4}}=170.022>A_{8}$, and $\frac{\left(A_{1}A_{3}A_{5}+A_{1}A_{3}A_{6}-A_{4}A_{6}\right)}{A_{4}}=1.11096<A_{7}$. Fig. 1c is the numerical simulation verifying the local stability criteria of the second predator free equilibrium point $E_{2}$. In the event that ecosystem parameters undergo substantial changes and stabilise at the quantities delineated above, the second predator is doomed to extinction on account of its comparatively slower conversion rate in contrast to the initial scenario. The value of the newly introduced parameter has an impact on the second predator population.

**Figure 2:**
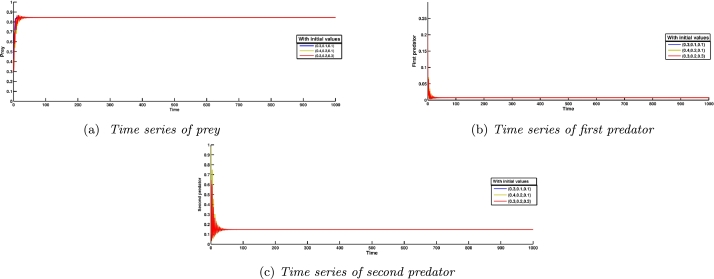
Local Stability of coexisting equilibrium *E*^⁎^(*x*^⁎^,*y*^⁎^,*z*^⁎^).

In an ecosystem, the most desirable outcome of a biosystem is the stability of its interior equilibrium. For the same reason, we consider parameter values as $A_{3}=2$, $A_{4}=0.4$, $A_{5}=4.536$, and the rest parameter values from Table 2, which satisfy the local stability conditions of the coexisting equilibrium point $E^{⁎}$ given by the Routh-Hurwitz criterion as $G_{11}=0.844371>0$, $G_{22}=1.96046>0$, $G_{33}=1.22722>0$, and $G_{11}G_{22}-G_{33}=0.428135>0$. A numerical simulation satisfying the abovementioned parameter values is shown in Figs. 2a, 2b, and 2c. It confirms the existence as well as local stability of the coexisting equilibrium point $E^{⁎}$ and hence verifies the theoretical results. Long-term conservation of population density is conceivable according to the biological interpretation, provided that all other system parameters remain constant and environmental parameters pertaining to the death rate of the first predator, consumption rate of the second predator, and consumption rate of the first predator are all equal to the aforementioned quantity.

**Figure 3:**
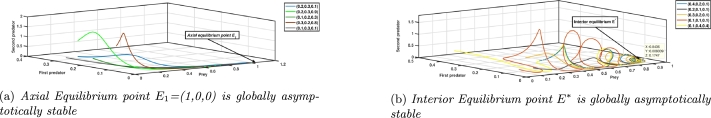
Phase portraits showing global stability of different equilibrium points.

Fig. 3 indicates that the axial equilibrium point as well as the cohabitation equilibrium point are globally asymptotically stable. As shown in Fig. 3b, starting from five different initial values, the solution trajectories of the system (3) eventually approach the interior fixed point $E^{⁎}$. All parameter values from the Table 2 are considered here. It confirms the global stability of the cohabitation equilibrium point $E^{⁎}$. Morever, Fig. 3a validates the global stability of the axial equilibrium point $E_{1}$. In this context, except for the values $A_{3}=0.875$, $A_{4}=0.0125$, and $A_{5}=4.536$, all other parameter values shown in the Table 2 are taken into consideration here.

### 5.2 Parameters affecting stability of equilibrium points

In this section, the impacts of different parameters on the stability of equilibrium points are analysed.

#### 5.2.1 Role of growth and death factors of both the predators

**Figure 4:**
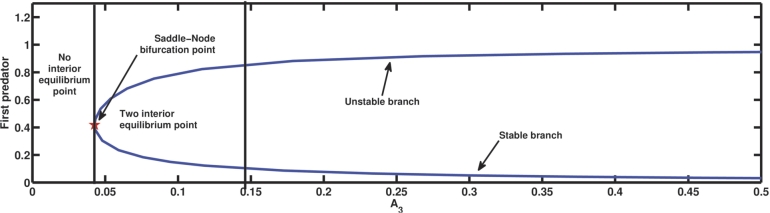
The emergence of saddle-node bifurcation for parameter *A*_3_.

$A_{3}$ is the parameter related to the death rate of the first predator. The mortality rate of a species is a critical determinant of its ability to persist. The Fig. 4 is drawn varying $A_{3}$ up to the value 0.5 and the rest of the parameter values from Table 2. It shows that the dynamical system experiences a saddle-node bifurcation at the coexisting equilibrium point when the parameter $A_{3}=A_{3}^{S}=0.04267$. When $A_{3}<A_{3}^{S}$ is employed, none of the coexisting equilibrium points are existent. However, when $A_{3}>A_{3}^{S}$, two simultaneous equilibrium points come to exist, one stable and another unstable, i.e., the coexisting equilibrium point with a relatively lower density of the first predator than the other equilibrium point becomes stable. It is noteworthy that reducing $A_{3}$ to $A_{3}=0.4$ with the rest of the parameter values from the Table 2 induces the extinction of the second predator as $A_{1}A_{3}=0.008<A_{4}$, $\frac{A_{4}A_{6}-A_{1}A_{3}A_{6}}{A_{1}A_{3}}=2.548<A_{5}$, $\frac{A_{1}A_{3}A_{4}A_{5}+A_{1}A_{3}A_{4}A_{6}-A_{4}^{2}A_{6}-A_{4}^{2}A_{7}}{A_{1}^{2}A_{3}^{2}-A_{1}A_{3}A_{4}}=229.91>A_{8}$, and $\frac{\left(A_{1}A_{3}A_{5}+A_{1}A_{3}A_{6}-A_{4}A_{6}\right)}{A_{4}}=0.03976<A_{7}$. So, the bistability of both the coexisting equilibrium as well as the second predator free equilibrium comes into existence. Even considering $A_{3}=1$ and rest parameter values from Table 2, it shows that all three species survive as $G_{11}=0.720216>0$, $G_{22}=2.43926>0$, $G_{33}=1.15537>0$, and $G_{11}G_{22}-G_{33}=0.601422>0$. But, taking $A_{3}=21$ along with the same other parameter values as before, not only the first predator vanishes but also the second predator faces extinction, as $A_{1}A_{3}=0.42>A_{4}$ and $A_{7}=4.546>A_{5}$. From a biological standpoint, the aforementioned situation serves as a compelling illustration of the impact of the mortality rate of the first predator. Below a specific threshold of first predator mortality, coexistence becomes unattainable, rendering it difficult for all three species to sustain long-term survival simultaneously. However, once the mortality rate of the first predator surpasses a specific threshold, it enables the coexistence of the three populations in two concurrent steady states, one of which is stable while the other is unstable.

**Figure 5:**
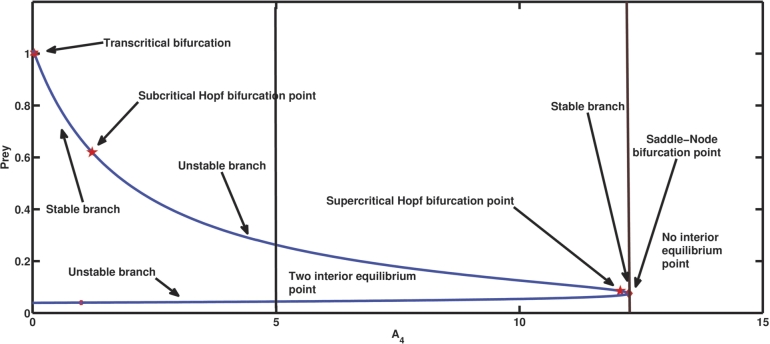
The appearance of subcritical Hopf bifurcation, supercritical Hopf bifurcation, saddle-node bifurcation, and transcritical bifurcation for parameter *A*_4_.

**Figure 6:**
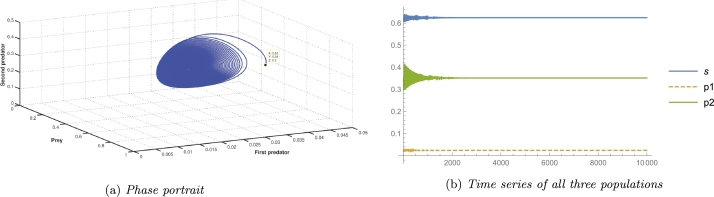
The illustration portrays the condition at $A_{4}<A_{4}^{H}$, denoting the phase prior to subcritical Hopf bifurcation at $A_{4}=A_{4}^{H}=1.2252$.

**Figure 7:**
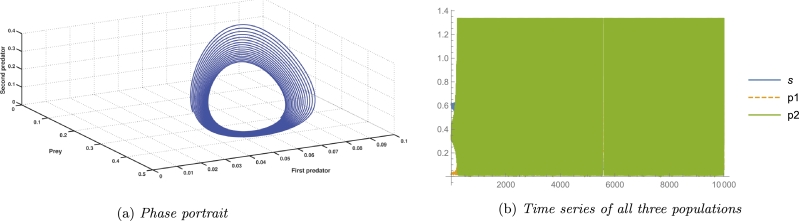
Depiction of the situation at $A_{4}>A_{4}^{H}$, which represents the state following a subcritical Hopf bifurcation at $A_{4}=A_{4}^{H}=1.2252$.

$A_{4}$ is the parameter related to the conversion efficiency or growth rate of the first predator. Fig. 5 depicts the progression of the coexisting equilibrium as $A_{4}$ increases while retaining the other parameter values from Table 2. At $A_{4}=0$, along with other parameter values from Table 2, at first, the first predator vanishes, which in turn results in the extinction of the second predator as $A_{1}A_{3}>A_{4}$ and $A_{7}=4.546>A_{5}$. One of the coexistent equilibrium points having comparatively high prey density becomes an attractive fixed point due to transcritical bifurcation at $A_{4}=A_{4}^{T}=0.04$. Considering $A_{4}=0.4$, coexistence of all three species is possible as $G_{11}=0.844371>0$, $G_{22}=1.96046>0$, $G_{33}=1.22722>0$, and $G_{11}G_{22}-G_{33}=0.428135>0$. On the further increase of the same parameter value, the coextant equilibrium point becomes unstable, and the system shows a subcritical Hopf bifurcation at the point $A_{4}=A_{4}^{H}=1.2252$ as $G_{11}(A_{4}^{H})=0.619461>0$, $G_{22}(A_{4}^{H})=5.01653>0$, $G_{33}(A_{4}^{H})=3.10759>0$, $G_{11}(A_{4}^{H})$ $G_{22}(A_{4}^{H})$-$G_{33}(A_{4}^{H})$=0, and $G_{11}(A_{4}^{H})G_{22}^{\prime }(A_{4}^{H})+G_{22}(A_{4}^{H})G_{11}^{\prime }(A_{4}^{H})-G_{33}^{\prime }(A_{4}^{H})=129.828\neq 0$. i.e., it satisfies the NASC (as specified in Theorem (9)) for the existence of Hopf bifurcation. Procedures mentioned by Kuznetsov [60] are employed to determine the characteristics and orientation of bifurcating periodic solutions for the specified values of parameters, and we get $g_{20}=-5.13961+2.74761i$, $g_{11}=0.693581-3.96867i$, and $g_{21}=10.3931+32.8495i$. The value of the first Lyapunov coefficient is 0.0971749>0. The positive value of the first Lyapunov coefficient indicates the subcritical nature of the Hopf bifurcation (as mentioned in theorem (10)). Biologically, it means that initially, $A_{4}^{H}-\epsilon <A_{4}<A_{4}^{H}$, where *ϵ* is a small quantity, the coextant population follows a spiral trajectory, to reach the coexisting equillibrium, which can be seen from Fig. 5. The initial population gets to the stable coexisting equilibrium point with the aid of an unstable limit cycle encircling it. The drawback of this unstable limit cycle is that any population that coexists with the population size outside of it will never be able to reach the equilibrium point when the original coexisting population is numerically simulated while maintaining the specified parameter values. Figure 6, Figure 7 depict the precise situation.

**Figure 8:**
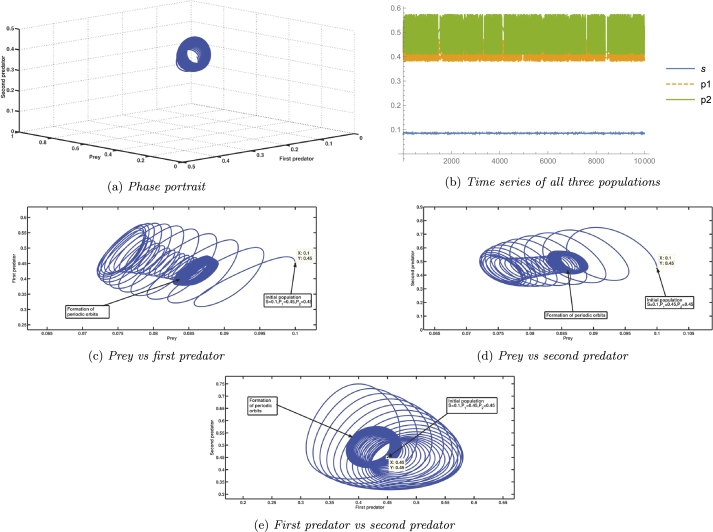
Illustration of the scenario at $A_{4}<A_{4}^{H⁎}$ (i.e., the state before super critical Hopf bifurcation at $A_{4}=A_{4}^{H⁎}=12.071019$.)

**Figure 9:**
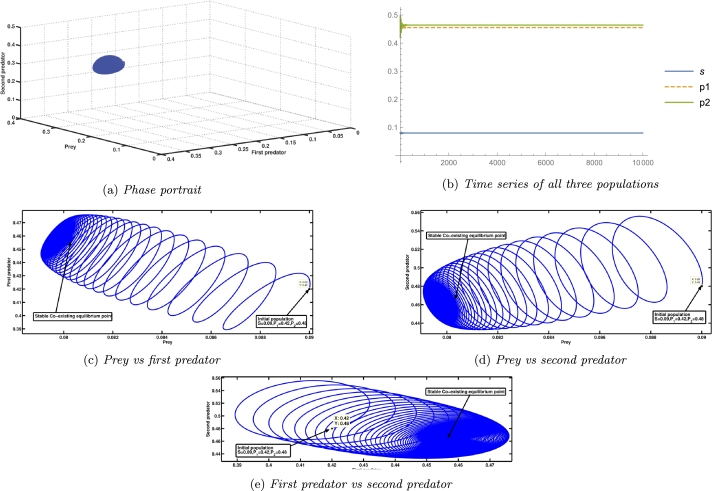
An illustration of the scenario at $A_{4}>A_{4}^{H⁎}$, which represents the state following a supercritical Hopf bifurcation at $A_{4}=A_{4}^{H⁎}=12.071019$.

On the further increase of the parameter $A_{4}$ (see Fig. 5), at $A_{4}^{H⁎}$=12.071019, the system (3) undergoes another Hopf bifurcation at the equilibrium point, which is supercritical as it satisfies the NASC, $G_{11}(A_{4}^{H⁎})=0.0851308>0$, $G_{22}(A_{4}^{H⁎})=11.2832>0$, $G_{33}(A_{4}^{H⁎})=0.960551>0$, $G_{11}(A_{4}^{H⁎})$ $G_{22}(A_{4}^{H⁎})$-$G_{33}(A_{4}^{H⁎})$=0, and $G_{11}(A_{4}^{H⁎})$ $G_{22}^{\prime }$($A_{4}^{H⁎}$) +$G_{22}(A_{4}^{H⁎})$ $G_{11}^{\prime }$($A_{4}^{H⁎}$)-$G_{33}^{\prime }$($A_{4}^{H⁎}$)= 4.78344≠0. Also, we get $g_{20}=-4.07662+1.01508i$, $g_{11}=1.25344-4.4808i$, and $g_{21}=-9.67663+11.0429i$. Thus, after some calculation we get the value of the first Lyapunov coefficient = −2.30622<0. Therefore, according to theorem (10), it indicates the supercritical nature of the Hopf bifurcation. Figure 8, Figure 9 describe the exact situation. The coexisting equilibrium point ceases to exist after an increment of the parameter value $A_{4}=A_{4}^{S}=12.248$, which is the saddle-node bifurcation point. When $A_{4}=3$, the second predator species faces extinction, whereas other species survive as $A_{1}A_{3}=0.04<A_{4}$, $\frac{A_{4}A_{6}-A_{1}A_{3}A_{6}}{A_{1}A_{3}}=3.848<A_{5}$, $\frac{A_{1}A_{3}A_{4}A_{5}+A_{1}A_{3}A_{4}A_{6}-A_{4}^{2}A_{6}-A_{4}^{2}A_{7}}{A_{1}^{2}A_{3}^{2}-A_{1}A_{3}A_{4}}=344.86>A_{8}$, and $\frac{\left(A_{1}A_{3}A_{5}+A_{1}A_{3}A_{6}-A_{4}A_{6}\right)}{A_{4}}=0.00917333<A_{7}$. The prior numerical simulations effectively demonstrated the importance of the growth rate of the first predator in the system (3). In the absence of population growth, the first predator becomes extinct, subsequently leading to the collapse of the second predator population. When the growth rate of the first predator increases steadily, a point is reached where cohabitation of all three species becomes feasible. At a certain growth rate of the first predator, periodic oscillations in the populations of the three species emerge due to a Hopf bifurcation. Subsequently, another Hopf bifurcation at a higher growth rate stabilises these oscillations. Furthermore, a saddle-node bifurcation emerges at a particular growth rate value, which biologically represents the threshold value of the growth rate of the first predator, beyond which coexistence becomes unattainable.

**Figure 10:**
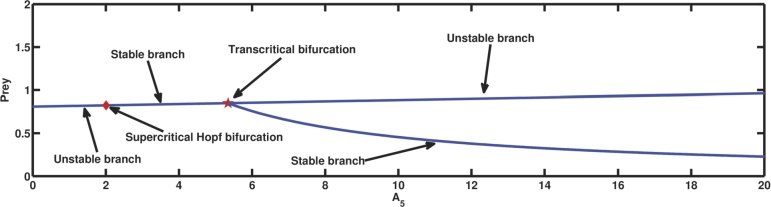
The occurrence of supercritical Hopf bifurcation and transcritical bifurcation for parameter *A*_5_.

Fig. 10 depicts the effect of $A_{5}$ (related to the conversion efficiency or growth rate of the second predator) on the coexisting equilibrium point as the said parameter is varied, keeping the rest of the parameter values as same as in Table 2. As $A_{5}$ is increased the unstable branch of the coexisting equilibrium point becomes stable through supercritical Hopf bifurcation occurring at $A_{5}=A_{5}^{H⁎}=2.007197$, and the equilibrium point gains stability as all the NASCs of the existence of Hopf bifurcation (as discussed in Theorem 9) are satisfied as $G_{11}(A_{5}^{H⁎})=0.825096>0$, $G_{22}(A_{5}^{H⁎})=5.83388>0$, $G_{33}(A_{5}^{H⁎})=4.81351>0$, $G_{11}(A_{5}^{H⁎})$ $G_{22}(A_{5}^{H⁎})$-$G_{33}(A_{5}^{H⁎})$= 0, and $G_{11}(A_{5}^{H⁎})$ $G_{22}^{\prime }$($A_{5}^{H⁎}$) +$G_{22}(A_{5}^{H⁎})$ $G_{11}^{\prime }$($A_{5}^{H⁎}$) -$G_{33}^{\prime }$($A_{5}^{H⁎}$) = -5.83715 ≠0 and we get $g_{20}=-11.8806+3.79617i$, $g_{11}=0.918258-12.3416i$, and $g_{21}=61.2278+195.711i$. Thus, the first Lyapunov coefficient is −0.190703<0 which indicates the supercritical nature of the Hopf bifurcation according to theorem (10). For example, at $A_{5}=4.536$, $G_{11}=0.844371>0$, $G_{22}=1.96046>0$, $G_{33}=1.22722>0$, and $G_{11}G_{22}-G_{33}=0.428135>0$ i.e., coexisting equilibrium is stable. The situation is accurately depicted by Figs. 13a, 13b, 14a, and 14b. However, through transcritical bifurcation, which occurs at $A_{5}=A_{5}^{T}=5.3449$, the stable branch becomes unstable again, but at the same time, another branch of the first predator free equilibrium point occurs with comparatively low prey density and gains stability. For instance, at $A_{5}=5.5$, the extinction of the first predator but the survival of the other two species is observed as $A_{5}=5.5>A_{7}$ and $\frac{A_{2}A_{5}^{2}-2A_{2}A_{5}A_{7}+A_{2}A_{7}^{2}+A_{3}A_{5}^{2}-A_{3}A_{5}A_{7}}{A_{5}A_{7}}=0.42153>A_{4}$. The growth rate of the second predator also has an impact on the aforementioned system (3). From an ecological perspective, it is intriguing to observe that a rise in the growth rate of the second predator has a positive influence on the population size of the prey species. Furthermore, it should be noted that at a specific threshold of the growth rate, a Hopf bifurcation takes place, leading to the emergence of periodic oscillations in the population dynamics of the three species. Moreover, the stability of the coexisting steady state undergoes a transition from stable to unstable when the growth rate of the second predator reaches a specific threshold. Consequently, the first predator becomes extinct within the system.

**Figure 11:**
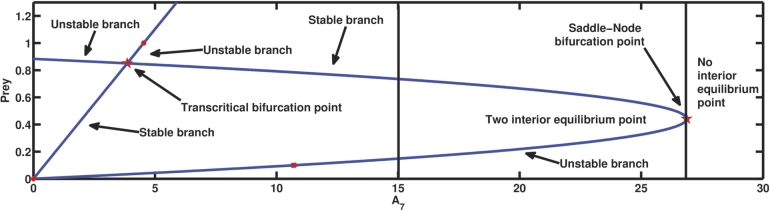
The manifestation of transcritical bifurcation and saddle-node bifurcation can be observed for parameter *A*_7_.

The parameter related to the mortality rate of the second predator is denoted by $A_{7}$. Fig. 11 illustrates the dynamical scenario of the system as $A_{7}$ is varied, keeping the rest of the parameter values as in Table 2. At $A_{7}=30$, the second predator becomes extinct as $A_{1}A_{3}=0.04<A_{4}$, $\frac{A_{4}A_{6}-A_{1}A_{3}A_{6}}{A_{1}A_{3}}=0.468<A_{5}$, $\frac{A_{1}A_{3}A_{4}A_{5}+A_{1}A_{3}A_{4}A_{6}-A_{4}^{2}A_{6}-A_{4}^{2}A_{7}}{A_{1}^{2}A_{3}^{2}-A_{1}A_{3}A_{4}}=328.813>A_{8}$, and $\frac{\left(A_{1}A_{3}A_{5}+A_{1}A_{3}A_{6}-A_{4}A_{6}\right)}{A_{4}}=0.4068<A_{7}$. However, at $A_{7}=26.869$, two interior equilibrium points appear through saddle-node bifurcation, of which the one with higher prey density gains stability, but as the said parameter value is decreased further, there is a transcritical bifurcation at $A_{7}=A_{7}^{T}=3.857$, which exchanges the stability of the said interior equilibrium point with the first predator free equilibrium point having comparatively low prey density. For example, reducing the death rate of the second predator to $A_{7}=1$, causes the first predator to become extinct as $A_{7}=1<A_{5}$ and $\frac{A_{2}A_{5}^{2}-2A_{2}A_{5}A_{7}+A_{2}A_{7}^{2}+A_{3}A_{5}^{2}-A_{3}A_{5}A_{7}}{A_{5}A_{7}}=7.20982>A_{4}$ which shows the stability of the first predator-free equilibrium. The significance of the mortality rate of the first predator, as previously examined, parallels the comparable importance of the mortality rate of the second predator. When the death rate exceeds a specific threshold for the second predator, it becomes impossible for all three species to live together. In this scenario, either one of the predators will disappear or only the prey will survive. Furthermore, the reduction in mortality rate of the second predator leads to the eventual extinction of the first predator.

#### 5.2.2 Role of kleptoparasitism in changing the qualitative scenario

In the biosystem (2), *a* is the parameter related to the rate of kleptoparasitism. When a→∞ then $A_{1}\to 0$, $A_{4}\to 0$, and $\lim_{a\to \infty }\frac{A_{4}}{A_{1}+P_{2}}=\lim_{a\to \infty }\frac{r_{2}r_{4}k}{(1+a)r_{1}}=0$ i.e., no growth of the first predator. In other words, when the rate of kleptoparasitism by the second predator is exceptionally high, the growth rate of the first predator declines to zero, and the species eventually disappears from the system. When a→0, then $A_{1}\to \infty$, $A_{4}\to \infty$, and $\lim_{a\to \infty }\frac{A_{4}}{A_{1}+P_{2}}=\lim_{a\to \infty }\frac{r_{2}r_{4}k}{(1+a)r_{1}}=\frac{r_{2}r_{4}k}{r_{1}}$, i.e., when the kleptoparasitism rate is negligible, the two predators can coexist.

$A_{8}$ is the parameter relating to the conversion rate of the second predator of the food received through kleptoparasitism. When $A_{8}=0$ and the rest parameter values from Table 2, then the second predator population continues to decline and disappears from the system after a certain period of time, whereas the first predator and prey species persist, having a population density of $E_{2}(0.1,0.9,0)$ through time. This is because $A_{1}A_{3}=0.04<A_{4}$, $\frac{A_{4}A_{6}-A_{1}A_{3}A_{6}}{A_{1}A_{3}}=0.468<A_{5}$, $\frac{A_{1}A_{3}A_{4}A_{5}+A_{1}A_{3}A_{4}A_{6}-A_{4}^{2}A_{6}-A_{4}^{2}A_{7}}{A_{1}^{2}A_{3}^{2}-A_{1}A_{3}A_{4}}=45.9911>A_{8}$, and $\frac{\left(A_{1}A_{3}A_{5}+A_{1}A_{3}A_{6}-A_{4}A_{6}\right)}{A_{4}}=0.4068<A_{7}$. As $A_{8}$ is increased, the birth of interior equilibrium occurs through saddle-node bifurcation at the bifurcation parameter value $A_{8}=A_{8}^{S}=10.2$. One of the equilibrium points becomes stable, while the other one is unstable. The equilibrium point $E_{2}$ still maintains stability, making the system bistable between second predator-free equilibrium and interior equilibrium. At $A_{8}=45.9726=A^{T}$, a transcritical bifuraction takes place, changing the stability of the coexisting equilibrium point and the second predator-free equilibrium point, and the system becomes bistable due to the local stability behaviour of the two interior equilibrium points. Based on the aforementioned numerical analysis, it can be deduced from a biological perspective that in cases where the second predator is unable to obtain sustenance through kleptoparasitism, its population undergoes a slow decline and eventually faces extinction over a prolonged period of time. The facilitation of coexistence among all three species can be achieved through a certain rate of food intake via kleptoparasitism.

### 5.3 Numerical simulation of bifurcation scenario of codimension two

In this section, we discuss the presence of codimension two bifurcations across different parameter values. The Figs. 15 demonstrate the same. In addition, the methodologies outlined by Kuznetsov [60], [61] are employed to compute the first and second Lyapunov coefficients, corroborating the findings obtained by graphical evaluation.

Fig. 15a shows the trajectory of the Hopf bifurcation curve when both parameters $A_{1}$ and $A_{5}$ are varied simultaneously. When $A_{5}$ is increased, a specific type of codimension two bifurcation, known as generalised Hopf bifurcation, takes place at the parameter value $\left(A_{5},A_{1})=(1.0216,0.1367\right)$. This results in a shift in the direction of the Hopf bifurcation from supercritical to subcritical. In order to validate this assertion, we proceed to compute the values of the first and second Lyapunov constants utilising the formulas that are provided by Kuznetsov [60]. Consequently, the value of the first Lyapunov coefficient ($l_{1}$)is 0, but the second Lyapunov coefficient ($l_{2}$) is found to be −14.2794≠0. Again, there is another generalized Hopf bifurcation at the parameter value $\left(A_{5},A_{1})=(1.4996,0.1045\right)$ showing a change in the direction of the Hopf bifurcation from subcritical to supercritical. This shift is indicated by the fact that the first Lyapunov coefficient ($l_{1}$) has a value of 0, while the second Lyapunov coefficient ($l_{2}$) is 8.29404≠0. Next, considering different parameters $A_{4}$ and $A_{2}$, a Hopf bifurcation curve is drawn in Fig. 15b, this curve is strictly increasing as $A_{4}$ is increased, and the existence of one generalized Hopf bifurcation point is seen, i.e., there is a change in the direction of Hopf bifurcation from supercritical to subcritical one as $A_{4}$ is increased. In this case, it is observed that the first Lyapunov coefficient ($l_{1}$) has a value of 0, while the second Lyapunov coefficient ($l_{2}$) is ≠0. In Fig. 15c, two generalized Hopf bifurcation points can be observed at the parameter values $\left(A_{5},A_{4})=(1.1230,0.2896\right)$ and $\left(A_{5},A_{4})=(3.8733,0.9586\right)$. These points are located along a rising Hopf bifurcation curve, which is obtained by varying both $A_{4}$ and $A_{5}$, with $A_{5}$ being represented on the abscissa, the parameter values $\left(A_{5},A_{4}\right)$ are set to (1.1230,0.2896), the first Lyapunov coefficient ($l_{1}$) is found to be 0, while the second Lyapunov coefficient ($l_{2}$) is determined to be −3.08156≠0. Similarly, for the parameter values $\left(A_{5},A_{4})=(3.8733,0.9586\right)$, the first Lyapunov coefficient ($l_{1}$) is 0, while the second Lyapunov coefficient ($l_{2}$) is 8.82884≠0. Correspondingly, the direction of Hopf bifurcation changes from subcritical to supercritical and then again to subcritical. As $A_{3},A_{4}$ are varied in Fig. 15d, the Hopf bifurcation curve again shows two generalized Hopf bifurcation points with the changes in the direction of Hopf bifurcation as explained in Fig. 15a. It is obviously observable from figures that regularity condition is satisfied for the Bautin bifurcation. In numerical observations, it is noted that at the points of generalised Hopf bifurcation, the first Lyapunov coefficients ($l_{1}$) are 0, while the second Lyapunov coefficients ($l_{2}$) have non-zero values. This provides empirical evidences supporting the graphical findings presented in Fig. 15. The Hopf bifurcation point plays a crucial role in determining the emergence or cessation of periodic behaviour within populations, as a result of variations in a certain ecological parameter. When two parameters are simultaneously altered, a collection of bifurcation points is obtained, which is represented by a curve known as a Hopf bifurcation curve. The understanding of the periodicity in the population at the neighbourhood of each point on the curve is determined by whether it exhibits a subcritical or supercritical nature. The Bautin bifurcation refers to the specific threshold parameter value at which a transition from a subcritical to a supercritical nature, or vice versa, happens within a population. In the parametric spaces of $A_{1}-A_{5}$, $A_{2}-A_{4}$, $A_{4}-A_{5}$, and $A_{3}-A_{4}$, the system (3) exhibits many instances of generalized Hopf bifurcation (Bautin bifurcation). These observations provide as evidence for a transition between different types of oscillatory behaviour within the population, as viewed from an ecological standpoint.

## 6 Conclusion

Kleptoparasitism or food stealing may serve as a viable adaptive strategy for animals in situations when food scarcity is prevalent in their natural environment or when the energy expenditure required for hunting is excessively high. Through the act of food stealing, individuals have the potential to efficiently preserve energy and resources that would otherwise be used in hunting or foraging efforts. Therefore, it can be argued that this particular strategy is highly advantageous for a species in acquiring energy resources. Although there exists a delicate balance, excessive proficiency in kleptoparasitism may provoke retaliatory aggression from the prey or result in a gradual deterioration of the kleptoparasite's own hunting abilities. There is a scarcity of scholarly literature pertaining to the mathematical modelling of kleptoparasitism, particularly in regard to the energy depletion resulting from the retaliatory behaviour of the predator (host) following kleptoparasitism. In this study, we aim to examine the impact of this factor on a system consisting of two predators and one prey.

We provided a model of two predators and one prey in the present paper, with Holling type-I functional responses for both predators. The two predator species engage in normal interspecific competition. Through kleptoparasitism, the first predator furnished the second predator with a supplementary food supply. Understanding the role of kleptoparasitism in influencing the population dynamics of the second predator as well as the first predator is one of the most intriguing aspects of this work. Obviously, the second predator is reliant on the first predator for its secondary food source. This paper extracts the dependency scenario of the second predator on the first predator.

The biosystem (3) demonstrates the possibility of five different types of ecologically feasible equilibrium points. A trivial equilibrium point that represents the absence of all species in the system is not the ideal situation from an ecological perspective. As both predators are specialist predators, the probable realisation of trivial equilibrium is the overeating of the prey by either or both predators, resulting in the extinction of the prey first, followed by the extinction of both predators owing to a lack of food source. Because the trivial equilibrium point is unstable, the long-term result most likely involves the continuous presence of prey species. Further, if both predators become extinct, the biosystem allows prey species to flourish up to their environmental carrying capacity. This is due to the stability of the axial equilibrium under certain parametric conditions. Stability conditions for the axial equilibrium point ecologically interpret that the likelihood of reaching the axial equilibrium point increases as the ratio of the first predator's growth rate to its death rate approaches a smaller value and as the second predator's death rate exceeds its growth rate. This is equally true from an ecological standpoint. Surprisingly, interspecific competition between the two predator species plays no role in driving a population trajectory to axial equilibrium.

The first boundary equilibrium point depicts a second predator-free system in which the first predator and prey survive but the second predator disappears. One of the necessary requirements for the stability of this equilibrium point is that the first predator's growth rate must be greater than its death rate. It can be seen from the stability conditions of this equilibrium point that the parameter relating to the second predator's loss in growth rate due to competition has a direct impact on this equilibrium's stability. It is intriguing to observe that the absence of the first predator because of competition has minimal impact on the stability of the second predator free equilibrium.

The second boundary equilibrium point depicts the first predator-free scenario, in which the second predator and prey species survive but the first predator becomes extinct. The growth rate of the second predator owing to the primary food resource should be greater than its own mortality rate, as the secondary growth rate due to kleptoparasitism will be ineffective due to the extinction of the first predator. Consequently, the second predator relies on a food source that is independent of the first predator in order to ensure its own existence.

An optimal outcome for a biosystem is characterised by the existence and stability of a coexisting equilibrium point. Ecologically, a coexisting equilibrium point depicts a scenario in which all of the species in a biosystem may coexist indefinitely. The existence and stability of the coexisting equilibrium point have been ascertained analytically and numerically in our model. It establishes the fact that in our system, all three species can coexist under certain conditions. If the first predator's growth rate is lower than its death rate, the first predator cannot survive because it lacks an alternate food source. Coexistence is therefore impossible.

The mortality rate of a species is critical to sustaining coexistence in a biosystem. When the per capita death rate of one species continues to rise, the equilibrium density of that species declines while the equilibrium density of its competitor increases [63]. Disease-related death, human-induced death, and other factors can all contribute to an increase in a predator's death rate. In this model, it can be seen that when the death rate of the second predator is less than its growth rate and the growth rate of the first predator is very low, the survival of the second predator depends entirely on its ability to hunt its prey, and kleptoparasitism doesn't help the second predator very much. However, if the second predator's death rate is higher than its growth rate, it will not be able to maintain its biomass through predation alone and will instead need to rely on kleptoparasitism, making it highly dependent on the first predator population. When the latter declines, the second predator's biomass will decline as well. Also, it is evident that at very high death rates for second predators, they cannot survive and hence vanish, as observed in this system. The occurrence of saddle-node bifurcation for $A_{7}$, which can be seen in Fig. 11, provides the highest threshold value of $A_{7}$ under the parametric conditions set in Table 2 beyond which coexistence is impossible. However, below this threshold, kleptoparasitism contributes to the maintenance of coexisting equilibrium. When the second predator's death rate is very low and the growth rate is greater than the death rate, then both predators' survival is dependent on their functional responses, and thus, due to an increase in interspecific competition between the predators, the first predator vanishes from this system, triggering a transcritical bifurcation for the parameter $A_{7}$ as shown in Fig. 11, changing the stability of the coexisting equilibrium point $E^{⁎}$, and as a result, $E^{⁎}$ becomes unstable and $E_{3}$ becomes stable. When the death rate of the first predator i.e., $A_{3}$, is sufficiently high, the system undergoes a transcritical bifurcation, which changes the stability of the equilibrium points $E^{⁎}$ and makes it unstable, and the axial equilibrium point $E_{1}$ becomes stable, which means ecologically that the first predator will vanish, but along with it, the second predator will also become extinct. However, when the first predator's death rate ($A_{3}$) is exceedingly low, a saddle-node bifurcation occurs in the system, as shown in Fig. 4, indicating the minimal threshold value of $A_{3}$ below which coexistence of all three species becomes unfeasible. This represents the scenario in which the first predator overeats prey, and as a consequence, the ecosystem either collapses or reaches axial equilibrium.

Hopf bifurcation does not occur around the equilibrium points $E_{1},E_{2},E_{3}$, implying that periodic oscillations in population dynamics do not occur when one or both predators become extinct.

Many occurrences of generalised Hopf bifurcation are found in this system, which can be seen as shown in Fig. 15. Using the generalised Hopf bifurcation, the second Lyapunov coefficient can be used to calculate the relative position of the stable and unstable limit cycles.

A species' growth rate is also a significant factor in determining its long-term survival. In our biosystem, $A_{4}$ is related to the first predator's growth rate. The Fig. 5 shows that for a very low value of $A_{4}$, if the mortality rate of the second predator is greater than its growth rate, the system undergoes a transcritical bifurcation in which coexisting equilibrium loses its stability and axial equilibrium becomes stable. At $A_{4}=1.2252$, the system goes through a subcritical Hopf bifurcation, in which a coexisting equilibrium changes its stability and goes from stable to unstable, which can be seen in Figs. 5, 6a, 6b, 7a, 7b. Subcritical Hopf bifurcation can be identified by the limit cycle that results from the bifurcation that is unstable and overlaps the steady state in parameter space. In this scenario, the three species populations reach the stable coexisting equilibrium point with a damped periodic oscillation, which is conceivable due to the attraction of the attractive equilibrium point and repelling limit cycle. From Figs. 5, 8a, 8b, 8c, 8d, 8e, 9a, 9b, 9c, 9d, 9e, it can be seen that at $A_{4}=12.071019$, a supercritical Hopf bifurcation occurs, changing the stability of the coexisting equilibrium point. As a result, the coexisting equilibrium point becomes stable until a saddle-node bifurcation occurs, giving the threshold maximum value of the parameter $A_{4}$, after which no coexisting equilibrium can be found. A reduction in the value of $A_{4}$ below the supercritical Hopf bifurcation point destabilises the steady coexisting equilibrium point, and the initial coexisting population begins to fluctuate periodically due to the birth of a stable limit cycle, and as the parameter decreases further, the diameter of the stable limit cycle rises. Due to the instability of the coexisting equilibrium point, any coextant population nearby the equilibrium point begins to fluctuate with a variable periodicity and, in the long run, attains a stable periodicity and continues to fluctuate with that fixed periodicity as the trajectory reaches the stable limit cycle. If any population starts its journey close to the stable limit cycle but outside of it, it will still reach the same level and reach a fluctuation with a definite period in the long run. Also, as the value of the parameter $A_{4}$ increases, the prey population continues to decline, the first predator gradually increases, and the second predator gradually diminishes until it vanishes from the system due to a transcritical bifurcation. The parameter $A_{5}$ is related to the second predator's growth rate from food gained via hunting prey. As the value of $A_{5}$ increases, the population of prey and the second predator continues to slowly increase, but the population of the first predator continues to decrease, and after a period of time, it disappears from the system as a result of a transcritical bifurcation as shown in Figs. 10. A supercritical Hopf bifurcation occurs at $A_{5}=2.007197$, and the value of the first Lyapunov coefficient becomes negative. It shifts the stability of the coexisting equilibrium point to produce a stable periodic oscillation in the population of all three species in the long term, which can be seen from Figs. 10, 13, 14.

**Figure 12:**
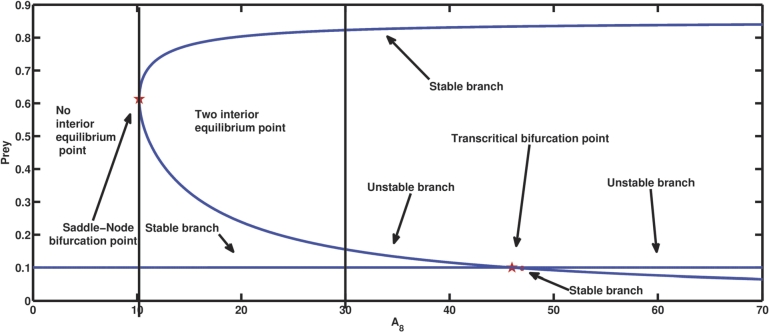
The equilibrium curves of two interior equilibrium alongwith the second predator-free equilibrium. It also depicts the emergence of saddle-node bifurcation and transcritical bifurcation with respect to parameter *A*_8_.

One of the important feeding strategies of a predator is kleptoparasitism. Kleptoparasitism plays a crucial role in sustaining coexistence in this system. Kleptoparasitism is critical to maintaining coexistence in this system. When the rate of kleptoparasitism (*a*) by the second predator is high, it can be seen that the growth rate of the first predator goes to zero, which is what happens in nature. Due to the high kleptoparasitism rate of the second predator, practically every prey killed by the first predator is stolen by the second predator, leaving nothing for the first predator to devour, and since predation is the only way for the first predator to survive, the growth rate becomes zero. Consequently, the first predator species becomes extinct. Now, if the death rate exceeds the growth rate of the second predator, it perishes alongside the first predator, and the axial equilibrium point becomes stable. However, if the growth rate exceeds the death rate of the second predator, it can coexist with the prey species, and the equilibrium point $E_{3}$ becomes stable. When the rate of kelptoparasitism is very low, i.e., when nearly no food of the first predator is stolen by the second predator, then $\lim_{a\to 0}A_{8}=0$, indicating that kleptoparasitism does not contribute much to the second predator's growth rate, and so the survival of both predator species is dependent on their functional responses. Thus, depending on the rates of interspecific competition, cohabitation between all three species is conceivable. These scenarios are highly typical from an ecological standpoint, proving that our model is very accurate in describing these specific ecological scenarios. Here, the parameter $A_{8}$ is related to the growth rate of the second predator due to the food obtained by kleptoparasitism. The appearance of saddle-node bifurcation at $A_{8}=10.20$ yields the lower-limit of this parameter below which coexistence is impossible as shown in Figs. 12. In a nutshell, it is clear from the dynamical scenario of the model (3) discussed above that the kleptoparasitism growth-related parameter $A_{8}$ is crucial in determining the saddle-node and transcritical bifurcations of the coexisting equilibrium point, which in turn affects the stability of the interior equilibrium.

**Figure 13:**
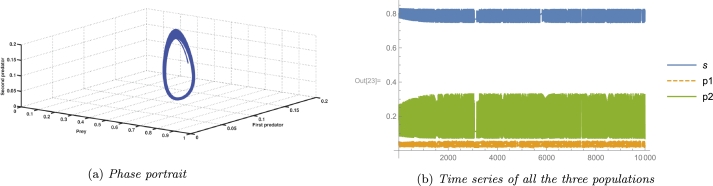
An illustration is provided for the scenario occurring at $A_{5}<A_{5}^{H⁎}$, which corresponds to the state prior to the occurrence of a supercritical Hopf bifurcation at $A_{5}=A_{5}^{H⁎}=2.007197$.

**Figure 14:**
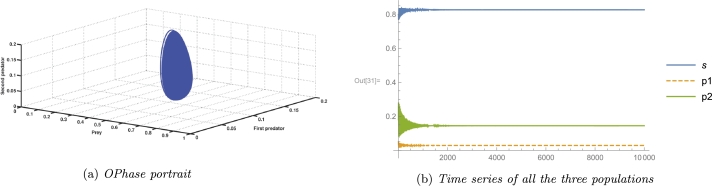
A depiction of the scenario occurring at $A_{5}>A_{5}^{H⁎}$, which denotes the state after a supercritical Hopf bifurcation at $A_{5}=A_{5}^{H⁎}=2.007197$.

**Figure 15:**
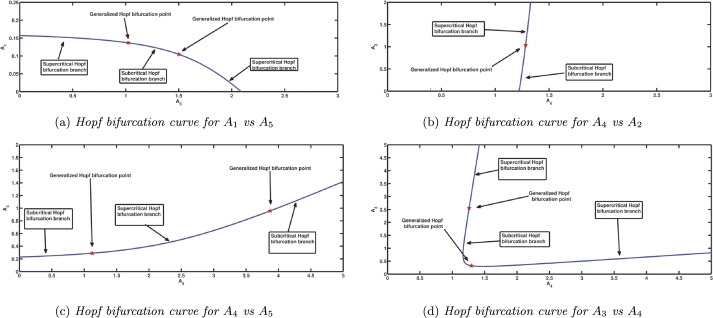
Emergence of generalized Hopf bifurcations.

## Funding

This research has not received any funding.

## CRediT authorship contribution statement

**Debasish Bhattacharjee:** Writing – review & editing, Writing – original draft, Visualization, Validation, Supervision, Software, Methodology, Investigation, Formal analysis, Data curation, Conceptualization. **Dipam Das:** Writing – review & editing, Writing – original draft, Visualization, Validation, Software, Methodology, Investigation, Formal analysis, Data curation, Conceptualization. **Santanu Acharjee:** Writing – review & editing, Writing – original draft, Visualization, Validation, Supervision, Conceptualization, Formal analysis, Investigation, Methodology. **Tarini Kumar Dutta:** Visualization, Validation, Supervision, Investigation, Conceptualization.

## Declaration of Competing Interest

The authors declare that they have no known competing financial interests or personal relationships that could have appeared to influence the work reported in this paper.

## Data Availability

No data was used for the research described in the article.
